# Soft-Pellet Feed Strategy for Broilers: Effects on Growth Performance and Liver–Intestinal Health

**DOI:** 10.3390/ani16152308

**Published:** 2026-07-26

**Authors:** Aixia Zhu, Wenjing Pan, Jiaqi Zhang, Shiqin Zhang, Chunwei Wang, Di Kong, Kai Peng, Fan Chen, Xin Yang

**Affiliations:** 1 Hubei Key Laboratory of Animal Nutrition and Feed Science, Wuhan Polytechnic University, Wuhan 430023, China; slgcx@whpu.edu.cn (A.Z.);; 2 Wuhan Livestock and Poultry Feed Engineering Technology Research Center, Wuhan Polytechnic University, Wuhan 430023, China; 3 Institute of Animal Science, Guangdong Academy of Agricultural Sciences, Guangzhou 510640, China

**Keywords:** feed type, growth performance, intestinal health, intestinal microbiota, soft-pellet feed

## Abstract

A total of 288 male Cobb broilers were randomly assigned to four dietary treatments: crumbled-pellet feed (CPF), hard-pellet feed (HPF), standard-nutrient soft-pellet feed (SPF), and low-nutrient SPF, each with eight replicates of nine birds. The 42-day trial was divided into stage 1 (days 1–21) and stage 2 (days 22–42). In stage 1, the broilers fed standard-nutrient SPF showed higher final body weight (FBW) and lower dry matter feed conversion ratio (DMFCR) than those fed CPF (*p* < 0.05), along with enhanced liver antioxidant capacity, reduced serum uric acid (UA) and creatinine (CREA), and improved intestinal morphology and disaccharidase activities compared with the CPF group. In stage 2, continued SPF feeding increased abdominal fat, serum total cholesterol (TC), and liver malondialdehyde (MDA) and reduced intestinal microbiota diversity (*p* < 0.05). In contrast, the broilers switched to HPF in stage 2 achieved growth performance comparable to that of the broilers fed SPF throughout both stages (TG3) but without health impairment. Low-nutrient SPF maintained growth despite reduced protein levels but increased dysbiosis risk. Under the conditions tested in this study, feeding standard-nutrient SPF in stage 1 followed by HPF in stage 2 appeared to be the most effective strategy among those tested, maximizing performance while preserving health. However, because the dietary formulations were intentionally adjusted to achieve the desired nutrient levels for each feed type, the observed effects reflect the combined influence of feed form and nutrient composition; further studies with iso-nutrient controls are needed to confirm the specific role of feed form.

## Simple Summary

In this study, we tested a new soft-pellet feed on 288 male broilers over a 42-day trial consisting of two growth stages (starter and grower) and compared its effects with conventional crumbled and hard-pelleted feeds on growth performance and health. We found that giving soft-pellet feed during the starter stage helped broilers to grow faster and improved their gut health. However, feeding it throughout the entire rearing period increased fat deposition and caused liver stress. Based on our study, a possible optimal strategy was to feed soft-pellet feed early, then switch to hard-pelleted feed later. This approach supported good growth while keeping the birds healthy. We also found that a low-protein version of soft-pellet feed maintained growth but had some negative effects on gut bacteria. These findings, under the conditions of this experiment, could offer a practical feeding option for poultry producers, although further validation under commercial conditions is recommended.

## 1. Introduction

The livestock and poultry industries are undergoing an accelerated transformation toward precision and intelligence, driving the feed classification system toward greater refinement and diversification [1,2]. In poultry farming, commercial feeds are primarily categorized into three types: mash, pellets, and crumbles [3,4,5]. Mash feed is low-cost and fine-particulate and is sometimes used for brooding chicks and laying hens [6]. However, its suitability remains a subject of debate [7,8]. Pelleted feed offers comprehensive nutrition and is widely used for growing poultry due to its physical uniformity and ease of handling, making it the mainstream choice in commercial production [9]. Crumbled feed, produced by crushing hard pellets, has a smaller particle size and is commonly used as a transitional feed during the brooding period [10].

Despite their widespread use, these feed types have notable limitations. Mash feed generates dust that can be inhaled, potentially causing respiratory diseases and impairing gastrointestinal development [11]. Pelleted and crumbled feeds may impair the developing digestive tract of young chicks by increasing the proportion of fine particles through mechanical compression during the pelleting process, which reduces the structural stimulation required for proper foregut (especially the gizzard) development and may lead to impaired gizzard development and reduced grinding function [12,13]. Furthermore, the high hardness of these feed forms may limit their softening and mechanical breakdown in the digestive tract, thereby shortening the effective duration of enzymatic action and ultimately restricting nutrient release and absorption [14]. Therefore, there is a practical need for a feed that is adapted to the physiological characteristics of young broilers, promoting healthy growth and high performance.

When the nutritional composition is constant, feed type can significantly affect growth and development because different processing technologies alter nutrient digestibility [6]. Developing new feed types through innovative processing is a viable strategy to improve efficiency. Our research group has developed a thermally processed soft-pellet feed (SPF) using a patented process (European Patent No. 3251523) [15]. Unlike conventional wet feed, which is produced by simply mixing dry feed with water to improve feed intake while reducing dust [16], and unlike fermented feed, which relies on microbial fermentation to moderately enhance nutrient availability and generate certain beneficial metabolites [17], SPF is produced by a cooking process that achieves high starch gelatinization (>75%) and protein denaturation without adding exogenous microbes or enzymes. It is characterized by low dust, low hardness, large ingredient size, and high nutrient availability. Currently, SPF has demonstrated favorable application effects in monogastric animals [18].

It was hypothesized that SPF would confer greater benefits during the starter phase of broilers by mitigating intestinal damage and promoting development owing to its soft texture and enhanced digestibility, and that these early advantages might carry over into later growth phases. We further hypothesized that a standard-nutrient SPF would outperform a low-nutrient SPF in supporting starter growth, and that switching from SPF to HPF in the grower phase would better preserve the early benefits while avoiding the negative metabolic effects associated with continuous SPF feeding throughout the entire production cycle. However, the unique digestive physiology and stage-specific nutritional requirements of broilers may result in differential efficacy of SPF across growth phases. Therefore, this study compared the effects of various feed types (including SPF) on the growth performance, liver health, and intestinal health of broilers at different stages. The aim was to identify feed types that enhance both health and production efficiency and to provide a theoretical basis for stage-specific feed selection.

## 2. Materials and Methods

### 2.1. Ethics Approval

The study’s procedures complied with animal care and use guidelines and received approval (No. WPU202307012) from the Animal Welfare Ethics Committee of Wuhan Polytechnic University, Wuhan, China.

### 2.2. Experimental Design

**Figure 1:**
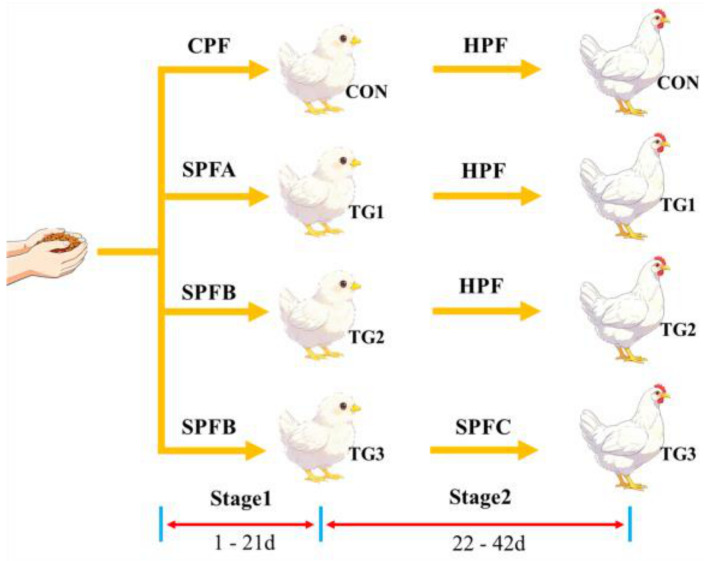
Feeding protocols of different dietary treatments across growth stages (CPF: crumbled-pellet feed, SPFA: soft-pelleted feed A, SPFB: soft-pelleted feed B, SPFC: soft-pelleted feed C, HPF: hard-pelleted feed, CON: control group, TG1: treatment group 1, TG2: treatment group 2, and TG3: treatment group 3).

Four dietary treatments were tested across two stages: stage 1 (days 1–21) and stage 2 (22–42 d). In stage 1, the control group (CON) received crumbled-pellet feed (CPF, standard nutrient). Treatment group 1 (TG1) received a low-nutrient SPF (SPFA), which had the same formulation as CPF but with higher moisture (20% higher) and consequently lower nutrient density. Treatment groups 2 and 3 (TG2 and TG3) received a standard-nutrient SPF (SPFB), formulated to match the nutrient levels of CPF at the higher moisture content. In stage 2, CON, TG1 and TG2 were switched to hard-pellet feed (HPF, standard nutrient). TG3 continued with a standard-nutrient SPF (SPFC), formulated to match the nutrient levels of HPF at the higher moisture content. The feeding protocol is illustrated in Figure 1 and summarized in Table 1.

### 2.3. Birds and Management

A total of 288 1-day-old healthy male Cobb 500 white-feather broilers (Xiaogan Anlu Zhengda Feed Business Department, Hubei, China) with the same genetic background and from the same hatch batch, having an average body weight of 47.07 ± 0.8 g, were selected. Birds were randomly assigned to one control group (CON) and three treatment groups (TG1, TG2, and TG3) using a computer-generated randomization list, with 8 replicates per treatment and 9 birds per replicate.

Prior to the experiment, the three-story poultry house was thoroughly disinfected. Throughout the experimental period, the ambient temperature was gradually reduced by 2–3 °C weekly, starting from 35 °C in the first week and eventually maintained at approximately 24 °C. The broilers were provided with a 16-h photoperiod and had ad libitum access to feed and water. Mortality was recorded daily, and feed intake was adjusted for dead birds by subtracting the feed allocated to the pen before death. Body weight was measured on days 1, 21, and 42 for growth performance calculations. At two stages (18–20 days and 39–41 days), feces were collected twice daily for three consecutive days per stage, with each replicate treated as an experimental unit, and stored at −20 °C for subsequent digestibility analysis. The study was conducted at the broiler experimental facility of Wuhan Polytechnic University, Wuhan, China.

### 2.4. Feed Preparation and Composition

The CPF was prepared by weighing the ingredients as per the formulation, adding an appropriate amount of water, extruding the mixture into pellets using a twin-screw extruder (model SZLH200, Jiangsu Zhengchang Group Co., Ltd., Liyang, China), and then air-drying the pellets to produce HPF. The CPF was obtained by crushing the HPF. The SPF was prepared by mixing all ingredients according to the formulation as per the method detailed in our team’s European patent (No. EP3251523A1, Supplementary Material S1). The SPF’s moisture content was generally about 20% higher than that of the pellet feed. The CPF shared the same formulation as SPFA; however, SPFA had a higher moisture content, which led to a lower nutrient level. The formulations of SPFB and SPFC were adjusted to ensure that their nutrient levels, despite higher moisture content, matched those of CPF and HPF, respectively. Feed nutrient contents were formulated to meet or exceed the nutrient requirements specified in the Chinese national standard for broiler compound feed (GB/T 5916-2020) [19] and Cobb 500 broiler recommendations [20] (Table 2). For clarity, SPFA was termed “low-nutrient SPF”, while SPFB and SPFC were collectively termed “standard-nutrient SPF”. After preparation, all experimental feeds were stored at −20 °C until use.

### 2.5. Growth Performance and Sample Collection Procedures

Feed intake was recorded daily, and broilers in each group were weighed using an electronic scale (CHS-D, Changzhou Leqi Electronic Technology Co., Ltd., Changzhou, China) on days 1, 21, and 42. Metrics such as final body weight (FBW), average daily feed intake (ADFI), average daily gain (ADG), and dry matter feed conversion ratio (DMFCR) were evaluated. On days 21 and 42 of the experiment, one broiler with a body weight approximating the mean of its respective replicate was selected for euthanasia (cervical dislocation following anesthesia) and sample collection. The sampling process involved collecting 4 mL of blood from the wing vein and transferring it to a blood collection tube. The tube was placed on ice to clot for serum separation and centrifuged at 3000 r/min for 10 min on a high-speed centrifuge (Neofuge 15R, Lykang Bio-Medical Technology Holding Co., Limited, Beijing, China), and the supernatant was divided and stored in a refrigerator at −80 °C (DW-86L626, Haier Group Co., LTD., Qingdao, China) for the detection of serum indicators. Subsequently, the liver and intestinal tract were dissected from each bird. The length of each intestinal segment (duodenum, jejunum, ileum, and caecum) was measured using a standard ruler (to the nearest 0.1 cm) after gently stretching the tissue on a flat surface without tension. Liver tissue samples and mucosal scrapings from the duodenum, jejunum, and ileum were collected into cryovials, snap-frozen in liquid nitrogen, and stored at −80 °C to evaluate hepatic antioxidant capacity and intestinal disaccharidase activities. Three-centimeter segments were excised from the mid-portions of the duodenum, jejunum, ileum, and cecum (avoiding the previously sampled mucosal sites) and fixed in 4% paraformaldehyde for histological examination of intestinal morphology. Cecal contents were aseptically collected into sterile 2 mL cryovials, snap-frozen in liquid nitrogen, and stored at −80 °C for subsequent analysis of the intestinal microbiota. Finally, slaughter performance metrics were determined using the methodology outlined by Yang et al. (2025) [21].

### 2.6. Approximate Composition and Nutrient Digestibility

The determination of proximate composition in feeds and feces was conducted as described by Mahmoud et al. (2023) [22]. Nutrient apparent digestibility was assessed using hydrochloric acid insoluble ash (AIA) as an endogenous marker, as outlined by Massuquetto et al. (2020) [23].

### 2.7. Serum Parameters

Before detection, the serum samples were placed in a refrigerator at 4 °C for slow thawing. After the samples became clear and free of bubbles, they were loaded onto an automatic serum biochemical analyzer (BS-240Vet, Mindray Medical International Limited, Shenzhen, China). The corresponding reagent kits were used to detect the levels of total protein (TP, #140821007), total triglyceride (TG, #141721004), total cholesterol (TC, #141621006), glucose (Glu, #141521008), alanine aminotransferase (ALT, #140121005), aspartate aminotransferase (AST, #140221004), uric acid (UA, #141220013), and creatinine (CREA, #141121009) in the serum.

### 2.8. Antioxidant Capacity

The key detection indicators assessed were total antioxidant capacity (T-AOC, #A015-2-1), superoxide dismutase (SOD, #A001-3-2), glutathione peroxidase (GSH-Px, #A005-1-2), and malondialdehyde (MDA, #A003-1-2). Before detection, a portion of the liver was taken and accurately weighed using an electronic analytical balance (ES2085A, Tianjin Deante Sensor Technology Co., Ltd., Tianjin, China), then placed in an EP tube. Subsequently, pre-cooled homogenate was added to the EP tube at a ratio of 1:9 (tissue to homogenate). Mechanical homogenization was performed using a low-temperature tissue homogenizer. After homogenization, centrifugation was carried out, and, finally, the supernatant was aspirated. All procedures were followed as per the kit instructions. The kits were obtained from Nanjing Jiancheng Bioengineering Institute, Nanjing, China.

### 2.9. Histomorphology

Intestinal tissues preserved in fixative were processed into H&E-stained sections following the procedure outlined by Xia et al. (2022) [24] (performed by a technician blinded to treatment allocation) and then observed and analyzed under an optical microscope (Olympus BX-41TF, Tokyo, Japan) equipped with an imaging analysis system.

### 2.10. Digestive Enzyme Activity

The detection indicators mainly included maltase (#A082-3-1) and sucrase (#A082-2-1) in the duodenum, jejunum, and ileum. Before detection, a portion of the intestinal mucosa was taken and accurately weighed using an electronic analytical balance (ES2085A, Tianjin Deante Sensor Technology Co., Ltd., Tianjin, China), then placed into an EP tube. Subsequently, pre-cooled homogenate was added to the EP tube at a ratio of tissue to homogenate of 1:9. Then, mechanical homogenization was performed using a low-temperature tissue homogenizer. After homogenization, centrifugation was carried out, and, finally, the supernatant was aspirated. All procedures were followed as per the kit instructions. The kits were obtained from Nanjing Jiancheng Bioengineering Institute, Nanjing, China.

### 2.11. Intestinal Microbiota

Broiler cecal content samples were sent to Majorbio Biopharm Technology Co., Ltd. (Shanghai, China). Total bacterial DNA was extracted using a commercial DNA extraction kit (FastPure Soil DNA Isolation Kit (Vazyme Biotech, Nanjing, China) (Magnetic bead), YH-soil). After purification, DNA concentration and purity were evaluated. A sequencing library was created and the V3-V4 hypervariable region of the bacterial 16S rRNA gene was sequenced using the Illumina MiSeq platform (San Diego, CA, USA) with the 338F/806R primer pair. Raw sequences were quality-filtered (Q > 20, no ambiguous bases) and trimmed. Rarefaction curves were generated to verify sufficient sequencing depth. Operational taxonomic units (OTUs) were clustered at 97% similarity using the RDP Classifier (version 2.11). Taxonomy was assigned using the SILVA database (version 138). For differential abundance analysis, the Benjamini–Hochberg false discovery rate (FDR) was applied, with q < 0.05 considered significant. The resulting raw sequencing data were processed using QIIME (version 2.0) software.

Because TG2 and TG3 received identical diets (SPFB) and management during the starter phase (stage 1), cecal samples from these two groups were pooled as a single treatment (standard-nutrient SPF, *n* = 8) for microbiota sequencing. In the grower phase (stage 2), TG2 (switched to HPF) and TG3 (continued SPFC) were analyzed separately as distinct treatments. This pooling strategy does not compromise the stage 1 comparison between CON, low-nutrient SPF (TG1), and standard-nutrient SPF (combined TG2/TG3).

### 2.12. Statistics and Analysis

Experimental data were organized using Microsoft Excel. One-way analysis of variance (ANOVA) was performed using SPSS software (version 26.0), followed by Duncan’s multiple range test for post hoc comparisons. GraphPad Prism (version 8.0) was used for figure generation. The pen was used as the experimental unit for all response variables (*n* = 8 per treatment). For serum biochemical parameters, liver antioxidant status, intestinal morphology, digestive enzyme activity, and intestinal microbiota, one bird per pen was sampled, and its measurements were considered representative of the pen mean for statistical analysis. Prior to statistical analysis, all data were assessed for normality using the Shapiro–Wilk test and for homogeneity of variances using Levene’s test. Statistical significance was set at *p* < 0.05. Data are presented as means with pooled standard error of the mean (SEM). No birds were excluded from the analysis, and no missing data occurred during the experiment.

## 3. Results

### 3.1. Effects on Growth and Slaughter Performance

At the end of stage 1, the final body weight (FBW) and ADG of the broilers in the CON group were the lowest, significantly lower than those in each treatment group (*p* < 0.05), while there were no significant differences among the treatment groups (*p* > 0.05) (Table 3). Meanwhile, the dry matter feed conversion ratio (DMFCR) of the broilers in the TG2 and TG3 groups was significantly lower than that in the CON and TG1 groups (*p* < 0.05). At the end of stage 2, the FBW and ADG of the broilers in the CON group were the lowest, significantly lower than those in each treatment group (*p* < 0.05), with the highest values in the TG3 group, followed by the TG2 group. Additionally, the DMFCR of the broilers in the TG3 group was the lowest, significantly lower than that in the other groups (*p* < 0.05).

**Figure 2:**
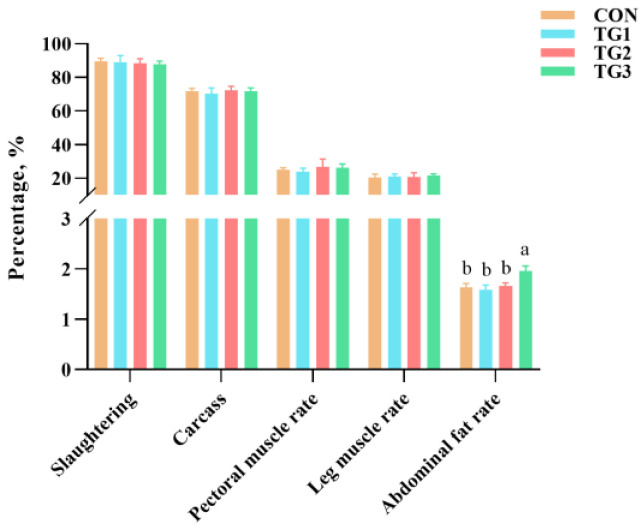
Effect of feed types on slaughter performance of broilers (*n* = 8). The differences in letters on the bars indicate a statistically significant difference (*p* < 0.05).

At the end of stage 2, no significant differences were observed in slaughtering, carcass, pectoral muscle rate, and leg muscle rate among the broiler groups. The TG3 group exhibited a significantly higher abdominal fat rate compared to the other groups (*p* < 0.05), with no significant differences observed among the remaining groups (*p* > 0.05) (Figure 2).

### 3.2. Effects on Digestibility of Nutrients

**Figure 3:**
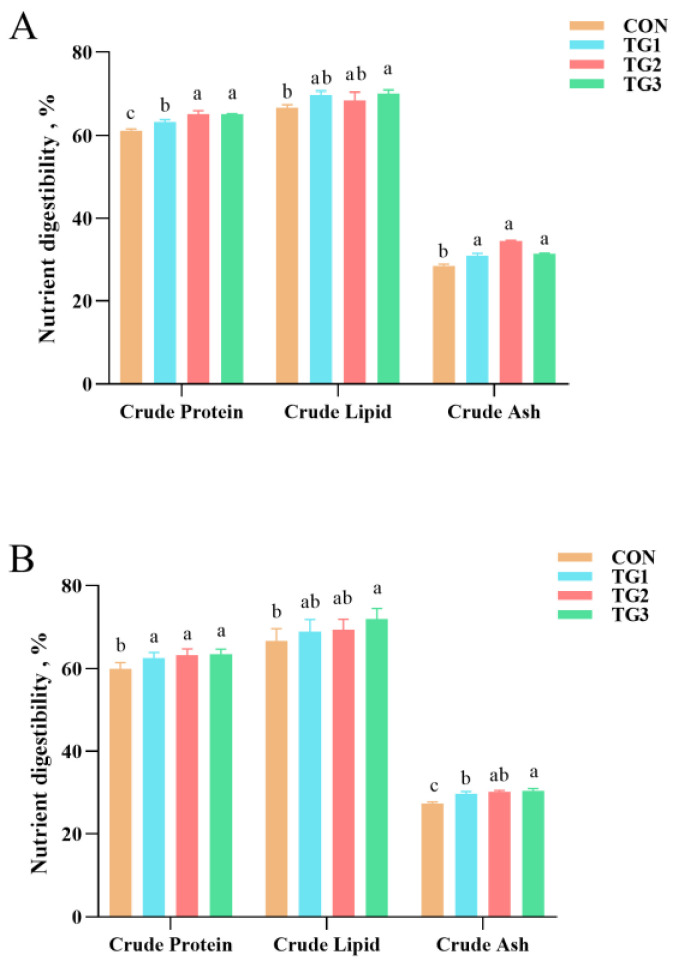
Effect of feed types on nutrient digestibility of broilers (*n* = 8). ((**A**) 9 birds per replicate in stage 1; (**B**) 8 birds per replicate in stage 2). Different letters on the bars indicate significant differences (*p* < 0.05).

In stage 1, the CON group exhibited significantly lower digestibility of crude protein, crude lipid, and crude ash compared to all the treatment groups (*p* < 0.05) (Figure 3A). In stage 2, the CON group’s feed exhibited the lowest digestibility levels for crude protein, crude lipid, and crude ash, significantly lower than those observed in the TG3 group (*p* < 0.05) (Figure 3B).

### 3.3. Effects on Serum Biochemistry and Liver Antioxidant Capacity

At the end of stage 1, the CON group demonstrated significantly elevated serum levels of UA and CREA in broilers compared to each treatment group (*p* < 0.05). Furthermore, its TP level was slightly lower than that of the TG1 group (*p* < 0.05). No significant differences were observed in the TC, TG, GLU, AST, and ALT levels among the groups (*p* > 0.05) (Table 4).

At the end of stage 2, the CON group exhibited the highest serum UA level, significantly higher than those in the TG1 and TG2 groups (*p* < 0.05), but showed no significant difference from the TG3 group (*p* > 0.05). Moreover, it had the lowest TP level, significantly lower than those in each treatment group (*p* < 0.05). The serum levels of TC, TG, GLU, AST, ALT, and CREA were the highest in the TG3 group, significantly higher than those in other groups (*p* < 0.05).

After the end of stage 1, the levels of T-AOC, SOD, GSH-Px, and MDA in the liver of the broilers in the CON group all reached the lowest values. Among them, the levels of SOD and GSH-Px were significantly lower than those in the TG2 and TG3 groups (*p* < 0.05) (Table 5). After the end of stage 2, the MDA level in the liver of the broilers in the CON group was also the lowest, significantly lower than that in the TG3 group (*p* < 0.05), with no significant difference compared to the TG1 and TG2 groups (*p* > 0.05). In broiler livers, the TG2 group exhibited significantly elevated levels of T-AOC, SOD, and GSH-Px compared to the CON group (*p* < 0.05).

### 3.4. Effects on Effects on Intestinal Tissue Morphology and Digestive Function

**Figure 4:**
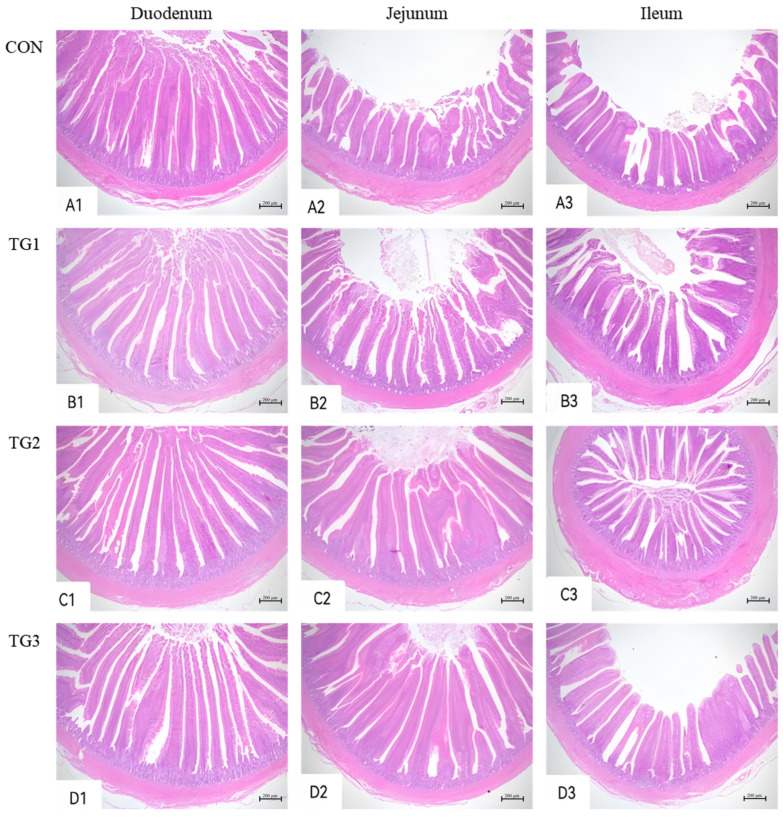
Effect of feed types on intestinal morphology of 21-day-old broilers. Intestinal sections were observed under a light microscope at 40× magnification. Scale bar: 200 μm. (**A1**,**B1**,**C1**,**D1**) duodenum; (**A2**,**B2**,**C2**,**D2**) jejunum; (**A3**,**B3**,**C3**,**D3**) ileum.

**Figure 5:**
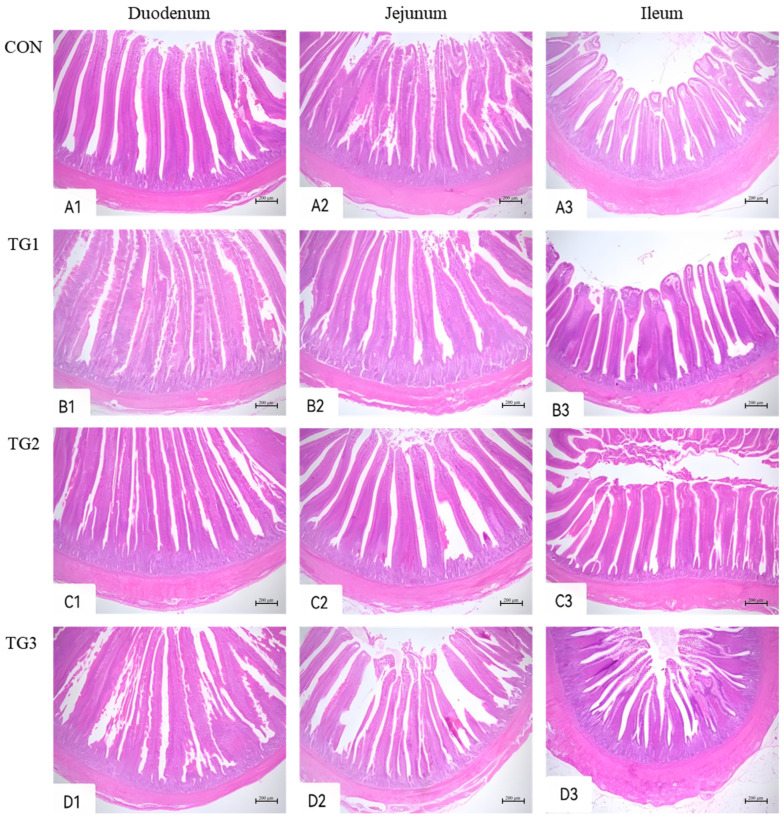
Effect of feed types on intestinal morphology of 42-day-old broilers. Intestinal sections were observed under a light microscope at 40× magnification. Scale bar: 200 μm. (**A1**,**B1**,**C1**,**D1**) duodenum; (**A2**,**B2**,**C2**,**D2**) jejunum; (**A3**,**B3**,**C3**,**D3**) ileum.

At the end of stage 1, all the groups exhibited no significant differences in duodenum length (*p* > 0.05). The jejunum, ileum, and cecum lengths in all the treatment groups exceeded those in the CON group, with the longest lengths observed in the TG2 and TG3 groups (Table 6). At the end of stage 2, no significant differences were observed in the lengths of the duodenum, jejunum, and cecum among the broiler groups (*p* > 0.05). The CON group exhibited the shortest ileum length in broilers, significantly less than the TG2 and TG3 groups (*p* < 0.05), with the TG2 group showing the longest ileum length. H&E staining was conducted on the intestinal tissues of the broilers from each group for observation. The results showed that different feed types did not cause structural damage to the intestinal tissues, with the intestinal villi arranged closely and no shedding or breakage observed (Figure 4 and Figure 5). Then, the morphology of the intestinal villi was measured and analyzed. The results indicate that the broilers in the CON group had the lowest villus height and V/C values in the duodenum, jejunum, and ileum at the end of each phase, significantly lower than those in the treatment groups (*p* < 0.05). No significant differences were observed among the treatment groups (*p* > 0.05), with the highest values recorded in the TG2 group (Table 7).

At the end of stage 1, the maltase and sucrase activities in the duodenum of the broilers showed no significant differences across all the groups (*p* > 0.05). The maltase and sucrase activities in the jejunum and ileum of the broilers were lowest in the CON group, significantly lower than in the other groups (*p* < 0.05), with no significant differences among the treatment groups (*p* > 0.05) (Table 8). At the end of stage 2, the broilers in the CON group exhibited the lowest maltase and sucrase activities in the duodenum, jejunum, and ileum, significantly lower than those in the other groups (*p* < 0.05), with the highest activities recorded in the TG2 group.

### 3.5. Effects on Intestinal Microbiota

**Figure 6:**
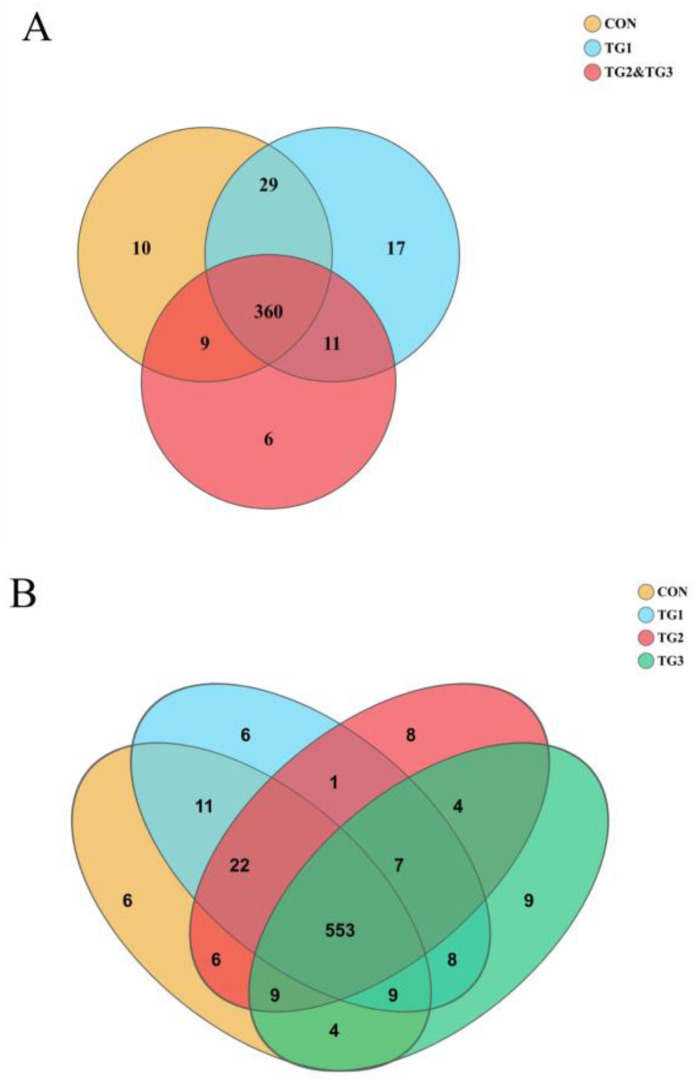
Effect of feed types on shared OTUs in cecum microbiota of broilers ((**A**) stage 1; (**B**) stage 2; *n* = 8). Numbers in the Venn diagram indicate OTU counts in each group and their overlaps.

**Figure 7:**
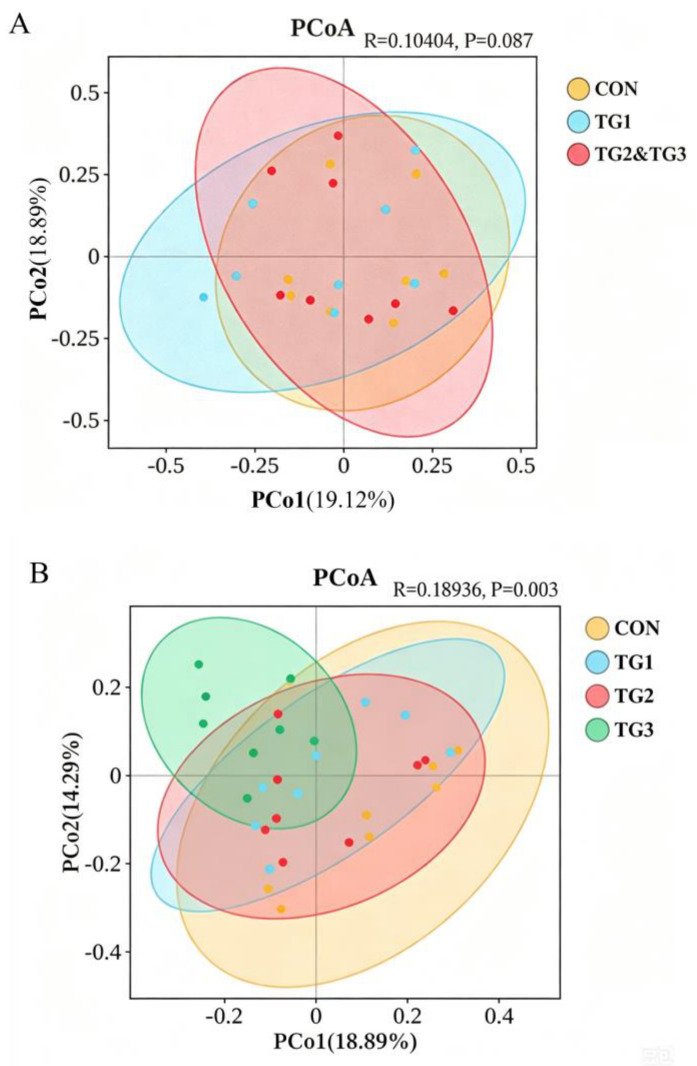
Effect of feed types on β-diversity in cecum microbiota of broilers ((**A**) stage 1; (**B**) stage 2; *n* = 8).

We performed 16S rRNA sequencing on cecal microorganisms of broilers at two growth stages to investigate how different feed types affect intestinal health. The Venn diagram illustrates the overlap and distinct elements among the datasets, highlighting both shared and unique components. Figure 6A,B show that, after the end of each stage, the number of unique OTUs in each group was relatively small, while the shared OTUs accounted for more than 80% of the total OTUs. The α-diversity analysis revealed no significant changes in the Ace, Chao1, Shannon, and Simpson indices among the groups after the end of stage 1 (Supplementary Table S1). After the end of stage 2 (Table 9), the CON group exhibited significantly higher Ace, Chao1, and Simpson indices compared to the TG3 group (*p* < 0.05). The β-diversity analysis indicated no significant differences in the microbial community structures among the groups after the end of stage 1 (*p* > 0.05) (Figure 7A). Significant changes in microbial community structures were observed in each group after the end of stage 2 (*p* < 0.05) (Figure 7B). The PERMANOVA analysis indicated that the TG3 group was the primary source of the observed changes.

**Figure 8:**
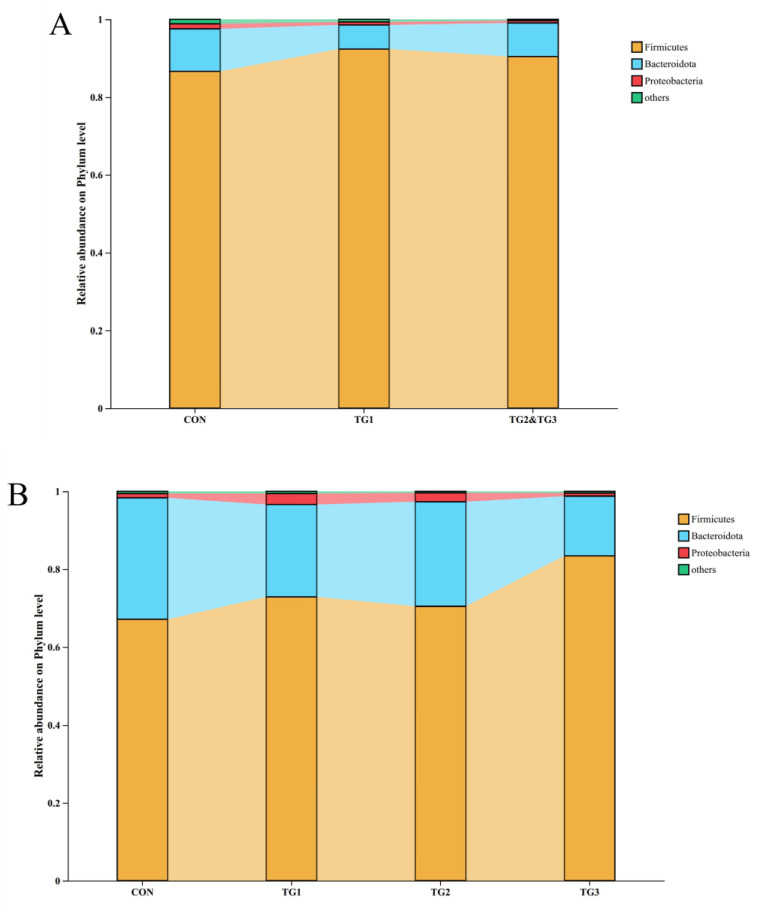
Effect of feed types on community composition at the phylum level in cecum microbiota of broilers ((**A**) stage 1; (**B**) stage 2; *n* = 8).

**Figure 9:**
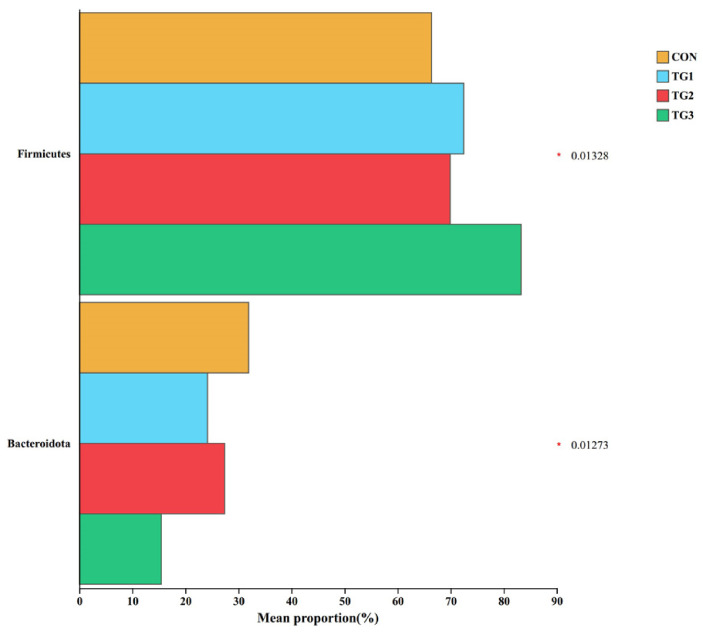
Effect of feed types on differentially abundant taxa at the phylum level in the cecal microbiota of the stage 2 broilers (*n* = 8). Asterisks (*) on the bars indicate significant differences (*p* < 0.05).

**Figure 10:**
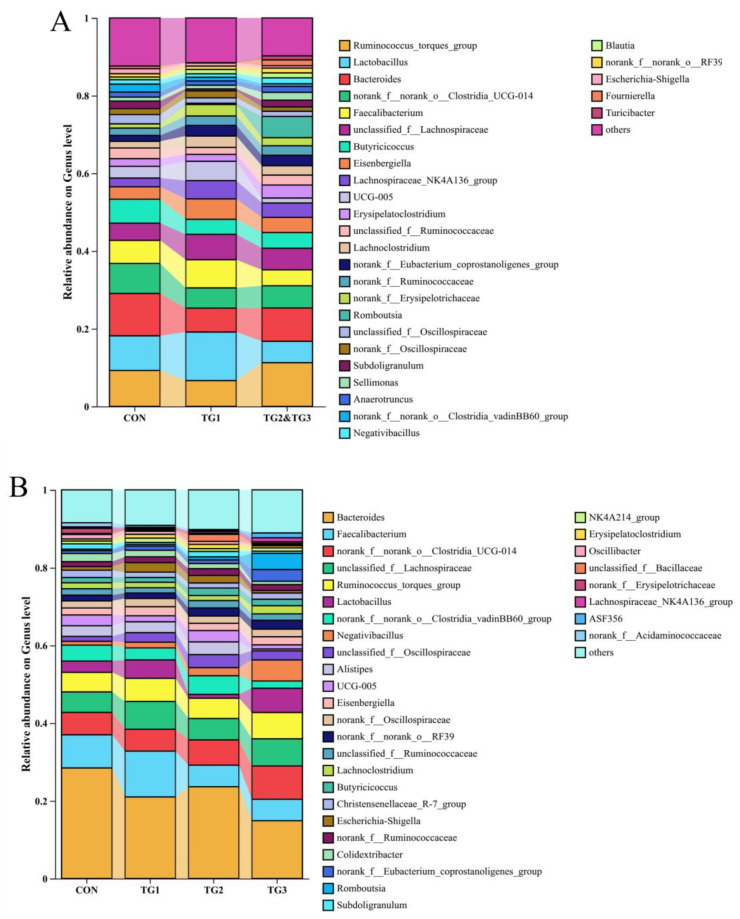
Effect of feed types on community composition at the genus level in cecum microbiota of broilers ((**A**) stage 1; (**B**) stage 2; *n* = 8).

**Figure 11:**
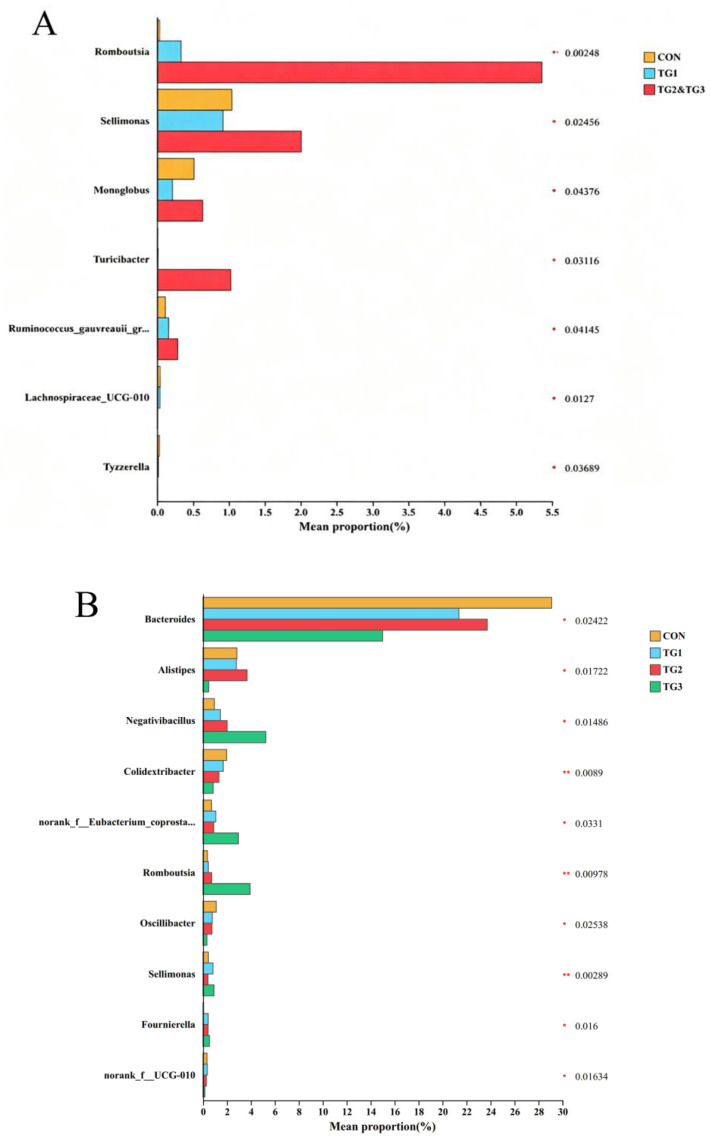
Effect of feed types on differentially abundant taxa at the genus level in the cecal microbiota of broilers ((**A**) stage 1; (**B**) stage 2; *n* = 8). Asterisks (*,**) on the bars indicate significant differences (*p* < 0.05).

Subsequently, the primary bacterial communities were analyzed at both the phylum and genus levels. At the phylum level, Firmicutes and Bacteroidota predominantly constituted over 95% of the intestinal microbiota in broilers across all the groups at the end of each stage, with Firmicutes being the most prevalent (Figure 8A,B). Moreover, after the end of breeding, Firmicutes abundance was notably greater in all the treatment groups compared to the CON group (*p* < 0.05) (Figure 9). At the genus level, after the end of each stage of breeding, the intestinal microorganisms of the broilers in each group were mainly composed of *Bacteroides*, *Faecalibacterium*, *norank_f_norank_o_Clostridia_UCG-014*, *unclassified_f_Lachnospiraceae*, *Ruminococcus_torques_group*, and *Lactobacillus* (Figure 10A,B). However, only *Bacteroides* showed significant differences among the groups after the end of stage 2 (*p* < 0.05) (Figure 11A,B).

**Figure 12:**
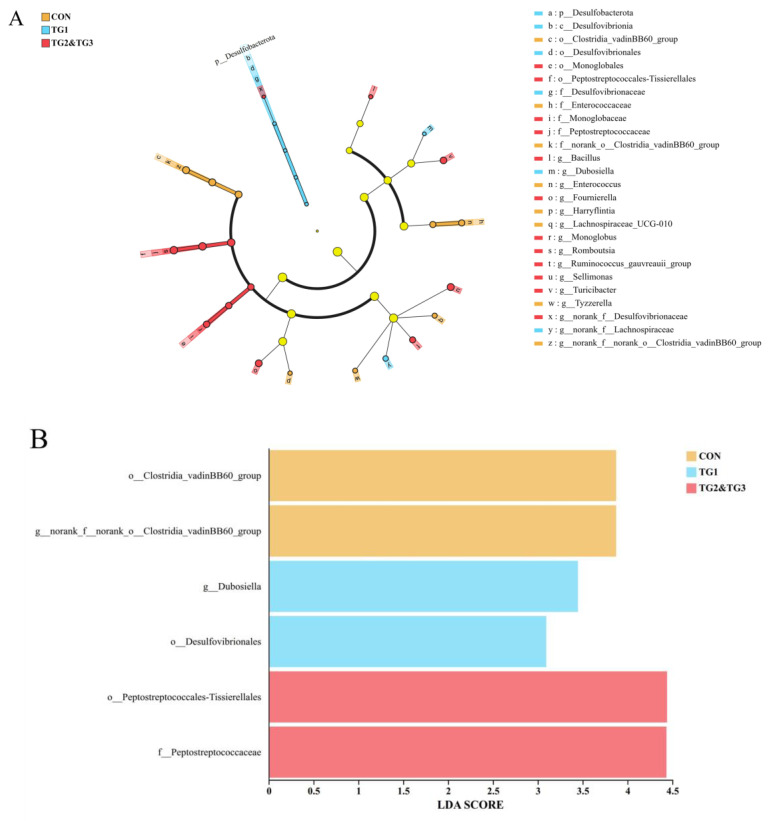
Effect of feed types on compositional differences in the cecal microbiota of stage 1 broilers ((**A**) LEfSe cladogram; (**B**) LDA score; *n* = 8).

**Figure 13:**
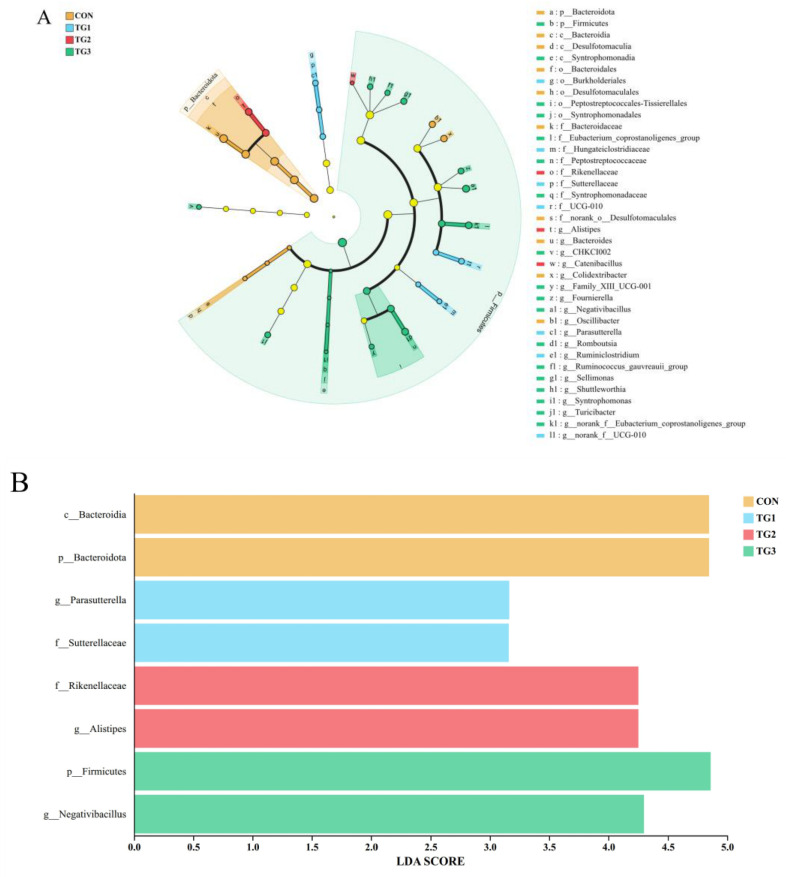
Effect of feed types on compositional differences in the cecal microbiota of stage 2 broilers ((**A**) LEfSe cladogram; (**B**) LDA score; *n* = 8).

Finally, through a LEfSe analysis, the two biomarkers with the greatest impact on species differences were screened from the phylum to genus levels in each group (LDA > 3, *p* < 0.05). After the end of stage 1, the two biomarkers in the CON group were Clostridia_vadinBB60_group and *norank_f_norank_o_Clostridia_vadinBB60_group*, those in the TG1 group were *Dubosiella* and Desulfovibrionales, and those in the TG2 and TG3 groups were Peptostreptococcales-Tissierellales and Peptostreptococcaceae (Figure 12A,B). After the end of stage 2, the two biomarkers in the CON group were Bacteroidia and Bacteroidota, those in the TG1 group were Parasutterella and Sutterellaceae, those in the TG2 group were Rikenellaceae and Alistipes, and those in the TG3 group were Firmicutes and *Negativibacillus* (Figure 13A,B).

## 4. Discussion

Feed processing technology is an important factor affecting feed quality [25]. Differences in farmed animals’ production performance across different feed types are primarily due to how different feed processing methods affect nutrient absorption and utilization efficiency. These internal changes are ultimately manifested as differences in production performance or health status [26,27,28]. In this study, broilers fed standard-nutrient SPF showed better FBW and DMFCR, with the TG3 group performing the best. This advantage was sustained throughout the entire feeding trial. We speculate that this result is likely due to the unique processing technology used in SPF preparation, which increases starch gelatinization and reduces both anti-nutritional factors and feed hardness [17], although we cannot fully exclude a contribution from the dietary formulation itself. These changes improve nutrient availability, ultimately leading to reduced feed consumption and enhanced growth and slaughter performance in broilers. Regarding the low-nutrient SPF, the results showed that, although the TG1 birds achieved growth performance that is comparable to TG2 and TG3 during stage 1, this was likely due to their substantially higher feed intake. This observation highlights the importance of maintaining adequate nutrient density in SPF formulations to avoid excessive feed intake and increased feed costs. Furthermore, it should be clarified that the growth performance of the broilers in all the treatment groups in this study was lower than that typically achieved in commercial broiler production. This does not diminish the comparative advantages observed among the dietary treatments tested. In fact, our diets were formulated only partially according to the Cobb 500 broiler management guide, and the actual nutrient densities were lower than those used in commercial Cobb 500 production. In addition, the experimental environment could not replicate the optimized conditions of commercial production systems. These factors likely contributed to the lower final body weights observed in this study. Slaughter performance is mainly used to reflect the mass proportion of different tissues and meat production performance [29]. The study found that only the abdominal fat rate in the broilers of the TG3 group was significantly higher compared to the other groups, with no notable changes in the other indicators. It should be noted that the broilers in the TG3 group received diets with the same nutrient levels as those in the TG2 group throughout the entire feeding period. Combined with the results of nutrient apparent digestibility, we speculate that the soft-pellet feed promotes fat digestion and absorption, thereby leading to increased abdominal fat deposition. An excessively high abdominal fat rate will have a negative impact on the body [30]. Future research should aim to optimize SPF formulation during the stage 2 feeding phase to fully realize its potential.

Serum biochemical indicators serve as markers of overall physiological status. Specifically, TP, TC, TG, and GLU are key indicators of nutrient metabolism [31,32,33], whereas ALT, AST, UA, and CREA reflect liver and kidney function, respectively [34,35]. In this study, the serum TP levels in the broilers from the TG1, TG2, and TG3 groups were generally higher than those in the CON group throughout the entire experimental period. This suggests that feeding SPF to chicks during stage 1 improves dietary protein absorption and utilization during this period [36]. This effect may be attributed to the role of SPF in promoting the development of the chicks’ digestive tract, as evidenced by the corresponding morphological data. In addition, compared with the other groups, the TC level in the TG3 group increased significantly only after the end of stage 2. This may also be attributed to the higher digestibility of dietary lipid, which led to lipid accumulation, oxidative stress, and subsequent liver damage [37], thereby elevating the serum ALT and AST levels. Interestingly, when the TG3 group was excluded from the comparison, after the end of stage 2, the levels of ALT, AST, UA, and CREA in the broilers from the TG1 and TG2 groups were lower than those in the CON group. These results suggest that feeding SPF during stage 1 may alleviate subsequent metabolic stress on the liver and kidneys.

In poultry, the liver is an essential organ for metabolism. It is susceptible to damage induced by oxidative stress, which can disrupt systemic metabolic balance and compromise organismal health [38,39]. T-AOC, SOD, GSH-Px, and MDA serve as key indicators for assessing antioxidant defense against oxidative damage [40]. In this study, after the end of stage 2, the broilers in the TG3 group exhibited significantly higher liver MDA levels than those in the TG2 group despite no significant difference in T-AOC. This indicates that the livers of the broilers in the TG3 group sustained oxidative damage. Notably, both the T-AOC and MDA levels were significantly higher in the TG3 group than in the CON group. This seemingly contradictory phenomenon may represent an adaptive compensatory mechanism [41] given that MDA is an end product of lipid peroxidation. This suggests that, although antioxidant capacity was elevated, it was ultimately insufficient to counteract the oxidative damage. A limitation of this study is that histological examination of the liver was not performed. Future studies should incorporate histopathological evaluation to confirm liver damage.

The intestine is the primary site for the body to absorb nutrients from food, and its developmental state, morphological integrity, and digestive capacity are closely related to its biological functions [42,43]. Intestinal length, villus morphology, and digestive enzyme activity serve as key indicators for assessing intestinal development, structural integrity, and digestive function, respectively [44,45,46]. The study found that the broilers in the TG1, TG2, and TG3 groups had longer duodenum, jejunum, ileum, and cecum lengths compared to the CON group, indicating that SPF consumption aids intestinal development and maturation [47]. The broilers in the TG1, TG2, and TG3 groups exhibited increased villus height, higher V/C ratios, and reduced crypt depth, suggesting that SPF consumption improves nutrient absorption and enhances the intestinal epithelial barrier [48,49]. Furthermore, the broilers in the TG1, TG2, and TG3 groups exhibited increased disaccharidase activity, enhancing nutrient absorption efficiency. The apparent digestibility of feed nutrients reflects the animal’s ability to absorb and utilize these nutrients [50]. In this study, the broilers fed SPF showed higher apparent nutrient digestibility than those fed CPF, providing direct evidence of improved nutrient utilization. Notably, SPF is more readily digestible than HPF. Under identical conditions, although the TG3 group exhibited lower disaccharidase activity than the TG2 group, it demonstrated higher apparent nutrient digestibility.

However, it remains unclear how the SPF promotes intestinal development and improves intestinal function. With the advances in intestinal microbiota research, increasing evidence suggests that feed can regulate the composition of the intestinal microbiota [51,52]. The intestinal microbiota’s composition and abundance are crucial for the digestive system’s development and function [53,54,55]. The number of OTUs directly reflects the microbial richness, and Venn diagrams illustrate the degree of similarity in microbial composition among samples [56,57]. The Ace and Chao1 indices reflect microbial richness, whereas the Shannon and Simpson indices reflect diversity [58]. In this study, the shared OTUs among the broilers in each group accounted for over 80% in both stages, indicating that different feed types did not cause significant changes in the composition of the intestinal microbiota. Further α-diversity analysis showed that, in stage 1, there were no obvious changes in the richness and diversity of the intestinal microbiota in the broilers of any group. It should be noted that, although TG2 and TG3 received the same diet during stage 1, this pooling design may limit the assessment of between-group variability to some extent. However, this was a deliberate design choice to avoid redundant sequencing and does not compromise the comparison among the three distinct treatments (CON, low-nutrient SPF, and standard-nutrient SPF) at this stage. However, during stage 2, the richness and diversity of the intestinal microbiota in the broilers of the TG3 group were lower than those in the CON group. These results imply that SPF affected the intestinal microecosystem of broilers during the later stage. The β-diversity analysis revealed a shift in the intestinal microbiota composition of the broilers in the TG3 group during stage 2.

To investigate the underlying causes of the observed differences in α- and β-diversity, we examined species composition at both the phylum and genus levels. At the phylum level, the intestinal microbiota of the broilers in each group predominantly consisted of Firmicutes and Bacteroidota throughout the entire experimental period, aligning with previous research [59,60]. Firmicutes and Bacteroidota, as dominant beneficial phyla, can prevent harmful bacterial colonization and improve intestinal function [61]. Additionally, after the farming period, the relative abundance values of Firmicutes in the TG1, TG2, and TG3 groups exceeded that in the CON group, mirroring the growth performance trends. This suggests that feeding SPF promotes the colonization of Firmicutes. Previous studies have suggested that Firmicutes and their metabolites are closely associated with feed digestion and absorption [62]. At the genus level, the dominant microbial communities in the intestinal tracts of the broilers in each group were generally similar throughout the entire experimental period, whereas more pronounced shifts were observed in the non-dominant microbial communities. During stage 1, Romboutsia, Sellimonas, Turicibacter, and Ruminococcus_gauvreauli_group may have played particularly important roles in the TG1, TG2, and TG3 groups as their changing trends were similar to the growth performance (Supplementary Figure S1). Similarly, during stage 2, Negativibacillus, norank_f_Eubacterium_coprostanoligenes_group, Romboutsia, and Fournierella may have played particularly important roles in the TG1, TG2, and TG3 groups (Supplementary Figure S2). It has been reported that Romboutsia, Sellimonas, Turicibacter, and Fournierella all have the ability to produce short-chain fatty acids and promote nutrient metabolism, which is beneficial to host growth [63,64,65]. There are few research reports on Ruminococcus_gauvreauii_group, Negativibacillus, and norank_f_Eubacterium_coprostanoligenes_group, which are potential beneficial bacteria, and their specific functions warrant further investigation. In conclusion, shifts in microbial communities at the phylum and genus levels during stage 2 account for the observed structural changes. However, it is noteworthy that, during stage 2, the TG1 and TG2 groups received the same feed as the CON group. This observation suggests that dietary differences during stage 1 may have had a lasting influence on the intestinal microbiota. We speculate that the early provision of SPF may contribute to the improved growth performance observed across the entire experimental period, but this inference requires confirmation in future studies with longer-term follow-up.

To pinpoint the key taxa exhibiting the highest discriminatory power among these changes, a LEfSe analysis was conducted. The results showed that, during stage 1, in the CON group, two biomarkers belonged to unclassified Clostridia (phylum Firmicutes). In the TG1 group, two biomarkers belonged to phyla Firmicutes and Proteobacteria, respectively. In the TG2 and TG3 groups, two biomarkers both belonged to the family Peptostreptococcaceae (phylum Firmicutes). During stage 2, in the CON and TG2 groups, two biomarkers both belonged to the phylum Bacteroidetes. In the TG1 group, two biomarkers both belonged to the order Burkholderiales (phylum Proteobacteria). In the TG3 group, two biomarkers both belonged to the phylum Firmicutes. Integrating the characteristics of the feed types at each stage with a growth performance analysis across the entire experimental period, the utilization of organic matter in CPF by chicks during stage 1 may require the assistance of Clostridia [66]. For the utilization of SPF with the same nutritional level, the assistance of Peptostreptococcaceae is needed to achieve better absorption efficiency [67] and to help maintain intestinal homeostasis [68]. It should be noted that, when chicks consumed low-nutrient SPF, the colonization of both potentially beneficial (inhibiting enteritis) [69] and potentially harmful (inducing inflammation) [70] bacterial communities increased. We speculate that this may reflect competition among bacterial communities in a specific intestinal environment, leading to no obvious phenotypic changes. The underlying mechanism warrants further investigation. During stage 2, the broilers in the CON and TG2 groups were switched to HPF. An increase in Bacteroidetes may aid in dietary fiber degradation and energy intake [71]. The broilers in the TG3 group still consumed SPF. The increased Firmicutes abundance established its dominance, enhancing energy harvest and promoting growth [72]. This may also have inhibited the colonization of other bacterial communities, thereby reducing α-diversity. The broilers in the TG1 group were switched to HPF, which seemed to increase the risk of enteritis [73]. This indicates that inadequate nutrition during the starter phase could elevate the likelihood of future diseases. Therefore, providing SPF with sufficient nutrients in stage 1 and HPF in stage 2 appears to be a more effective feeding strategy. Additionally, future optimization of the SPF formula used in stage 2 may further enhance growth performance without compromising broiler health. In conclusion, from a practical perspective, the optimal breeding strategy should take into account both production and health rather than merely focusing on maximizing production. Therefore, providing SPF with sufficient nutrients in stage 1 and HPF in stage 2 appears to be a more effective feeding strategy.

However, several limitations of this study should be acknowledged. First, the SPF formulation used in stage 2 has potential for further optimization, which may enhance growth performance without causing additional adverse effects. Second, the unique properties of the soft-pellet feed, including its higher moisture content and lower hardness, may pose significant challenges (such as microbial proliferation and product deterioration) for transportation and storage in future commercialization. Third, the observed effects reflect the combined influence of feed form and nutrient composition as the dietary formulations were intentionally adjusted to achieve the desired nutrient levels for each feed type; therefore, the contribution of each factor cannot be fully separated, although the findings may still offer useful insights into the commercial potential of this feed type. Fourth, the experiment was conducted using only male birds, a single 42-day production cycle, and one experimental facility; consequently, the findings may not be generalizable to other strains, sexes, rearing systems, or production conditions. Nevertheless, the relatively low overall growth performance achieved in this experiment should be taken into account when considering the direct applicability of our findings to modern commercial broiler production systems. Future studies should address these limitations through refined experimental designs to enable a more in-depth and accurate investigation of the underlying mechanisms.

## 5. Conclusions

In conclusion, this study systematically evaluated the effects of feeding SPF to broilers across both growth stages and identified an optimal feeding strategy: utilizing standard-nutrient SPF during stage 1 (1–21 d) and switching to HPF during stage 2 (22–42 d). This strategy not only enhances growth performance but also maintains health and carcass traits, thereby providing practical guidelines for feed management in the broiler industry.

## Figures and Tables

**Table 1:** Feeding regimens for broilers across whole stages.

Group	Stage 1	Stage 2	Moisture
CON	CPF (standard-nutrient) ^1^	HPF (standard-nutrient)	low
TG1	SPFA (low-nutrient) ^1^	HPF (standard-nutrient)	high
TG2	SPFB (standard-nutrient)	HPF (standard-nutrient)	high
TG3	SPFB (standard-nutrient)	SPFC (standard-nutrient)	high

The experimental design was conceived to compare practical feeding regimens combining feed form, nutrient density, and stage-specific strategy. The primary comparisons were: (i) CPF vs. standard-nutrient SPF in the starter phase (feed form effect, iso-nutrient); (ii) low-nutrient vs. standard-nutrient SPF (nutrient density effect); and (iii) continuous SPF vs. SPF→HPF (stage-specific strategy effect). We acknowledge that this approach does not fully isolate individual factors, but it reflects practical decision-making in commercial production. ^1^ Hard-pelleted feed (HPF) is defined as conventionally processed pellets with typical commercial hardness, in contrast to the softened texture of SPF.

**Table 2:** Formulation and proximate composition of different feed types (%, air-dry basis).

Items	CPF	SPFA	SPFB	HPF	SPFC
Ingredients ^1^					
Corn	59.15	59.15	48.59	62.2	54.54
Soybean meal	31.65	31.65	19.00	29.00	13.00
Corn protein powder	3.00	3.00	20.00	3.00	20.00
Dicalcium phosphate	1.60	1.60	2.00	1.10	1.50
Limestone	1.10	1.10	1.23	1.30	1.58
Salt	0.30	0.30	0.33	0.30	0.33
Soybean oil	2.00	2.00	7.00	2.00	7.00
L-lysine HCl	0.05	0.05	0.55	0.05	0.8
DL-methionine	0.15	0.15	0.1	0.05	0.05
Premix ^2^	1.00	1.00	1.20	1.00	1.20
Sum	100	100	100	100	100
Nutrient composition ^3^					
Crude protein (%)	20.59	16.29	20.78	19.57	19.70
ME (MJ/kg)	12.56	9.77	11.89	12.69	12.48
Calcium (%)	1.00	0.78	1.03	0.91	0.86
Available phosphorus	0.47	0.37	0.51	0.36	0.36
Methionine (%)	0.52	0.40	0.52	0.41	0.42
Lysine (%)	1.16	0.90	1.15	1.09	1.07

^1^ The feed formulation did not contain any bacteriostatic agents, antibiotics, or growth promoters. ^2^ The premix provided the following amounts per kilogram of complete diet: vitamin A 15,000 IU; vitamin D3 4500 IU; vitamin E 58.5 IU; vitamin K3 3 mg; vitamin B1 1.8 mg; vitamin B2 7.50 mg; vitamin B6 4.80 mg; vitamin B12 0.03 mg; biotin 0.24 mg; choline 600 mg; folic acid 1.20 mg; D-pantothenic acid 12.00 mg; niacin 45.00 mg; Cu 4 mg; Fe 42 mg; Mn 52.50 mg; Zn 45.50 mg; I 0.80 mg; Se 0.3 mg (1 to 21 days old); vitamin A 12,500 IU; vitamin D3 3750 IU; vitamin E 48.75 IU; vitamin K3 2.5 mg; vitamin B1 1.5 mg; vitamin B2 6.25 mg; vitamin B6 4 mg; vitamin B12 0.025 mg; biotin 0.2 mg; choline 480 mg; folic acid 1 mg; D-pantothenic acid 10.00 mg; niacin 37.50 mg; Cu 4 mg; Fe 42 mg; Mn 52.50 mg; Zn 45.50 mg; I 0.80 mg; Se 0.3 mg (22 to 42 days old). ^3^ Metabolic energy, methionine and lysine were the calculated values, while the others were all measured values.

**Table 3:** Effect of feed types on growth performance of broilers (*n* = 8).

Items	CON	TG1	TG2	TG3	SEM	*p*-Value
Stage 1 (1–21 d)						
FBW, g	623.19 ^b^	682.22 ^a^	686.80 ^a^	683.33 ^a^	6.41	<0.001
ADG, g	28.80 ^b^	31.76 ^a^	31.98 ^a^	31.82 ^a^	0.32	<0.001
ADFI, g	42.78 ^c^	62.54 ^a^	47.90 ^b^	46.46 ^ab^	1.45	<0.001
DMI, g	37.22 ^b^	43.78 ^a^	33.03 ^c^	32.52 ^c^	0.89	<0.001
DMFCR	1.29 ^a^	1.38 ^a^	1.04 ^b^	1.02 ^b^	0.03	<0.001
Stage 2 (22–42 d)						
FBW, g	1988.48 ^c^	2064.33 ^b^	2071.16 ^ab^	2134.00 ^a^	13.87	0.010
ADG, g	63.84 ^b^	64.28 ^ab^	64.36 ^ab^	67.52 ^a^	0.62	0.043
ADFI, g	145.89	151.59	148.18	149.64	2.02	0.804
DMI, g	126.92 ^a^	106.11 ^b^	103.73 ^b^	104.74 ^b^	2.26	<0.001
DMFCR	1.99 ^a^	2.05 ^a^	2.00 ^a^	1.61 ^b^	0.04	<0.001
Whole-stage (1–42 d)						
FBW, g	1988.48 ^c^	2064.33 ^b^	2071.16 ^ab^	2134.00 ^a^	13.87	0.010
ADG, g	46.74 ^c^	48.42 ^b^	48.57 ^b^	50.11 ^a^	0.33	0.002
ADFI, g	95.59 ^b^	108.15 ^a^	99.26 ^b^	99.31 ^b^	1.38	0.005
DMI, g	83.17 ^a^	75.71 ^b^	69.49 ^c^	69.51 ^c^	1.29	<0.001
DMFCR	1.65 ^a^	1.73 ^a^	1.50 ^b^	1.32 ^c^	0.03	<0.001
Mortality, %	8.33	6.94	8.33	6.94	1.26	0.964

^a–c^ Values within a row with different superscripts differ significantly at *p* < 0.05. Abbreviations: FBW, final body weight; ADG, average daily gain; ADFI, average daily feed intake; DMI, dry matter intake; DMFCR, dry matter feed conversion ratio; SEM, pooled standard error of the mean.

**Table 4:** Effect of feed types on serum biochemistry of broilers (*n* = 8).

Items	CON	TG1	TG2	TG3	SEM	*p*-Value
Stage 1 (1–21 d)						
TP, g/L	32.17 ^b^	33.80 ^a^	33.22 ^ab^	33.10 ^ab^	0.20	0.046
TC, nmol/L	158.58	159.93	167.15	164.09	2.91	0.290
TG, nmol/L	28.65	27.53	31.65	30.20	0.94	0.344
GLU, mg/L	214.76	204.99	225.36	217.33	3.74	0.206
AST, U/L	199.13	200.64	213.31	208.80	2.77	0.162
ALT, U/L	8.08	7.96	8.24	8.08	0.06	0.355
UA, μmol/L	241.28 ^a^	214.32 ^b^	210.10 ^b^	215.17 ^b^	4.15	0.008
CREA, mg/dL	53.15 ^a^	49.90 ^b^	48.52 ^b^	48.30 ^b^	0.59	0.042
Whole-stage (1–42 d) ^1^						
TP, g/L	38.09 ^b^	40.06 ^a^	39.66 ^a^	39.50 ^a^	0.24	0.021
TC, nmol/L	163.28 ^b^	160.05 ^b^	165.11 ^b^	188.73 ^a^	3.01	0.001
TG, nmol/L	31.27	29.98	31.21	33.03	0.86	0.679
GLU, mg/L	239.23	243.11	240.68	247.54	3.34	0.843
AST, U/L	244.970 ^b^	238.64 ^b^	237.01 ^b^	268.60 ^a^	2.90	<0.001
ALT, U/L	8.50 ^b^	8.28 ^c^	8.37 ^bc^	8.92 ^a^	0.05	<0.001
UA, μmol/L	256.22 ^a^	226.59 ^c^	230.39 ^bc^	249.86 ^ab^	3.91	0.009
CREA, mg/dL	54.39 ^b^	52.047 ^b^	54.03 ^b^	61.19 ^a^	1.02	0.004

^1^ Whole-stage (1–42 d) values were calculated based on the complete 42-day dataset rather than by combining or averaging the two stage-specific values. ^a–c^ Values within a row with different superscripts differ significantly at *p* < 0.05. Abbreviations: TP, total protein; TC, total cholesterol; TG, triglycerides; GLU, glucose; AST, aspartate aminotransferase; ALT, alanine aminotransferase; UA, uric acid; CREA, creatinine; SEM, pooled standard error of the mean.

**Table 5:** Effect of feed types on liver antioxidant capacity of broilers (*n* = 8).

Items	CON	TG1	TG2	TG3	SEM	*p* Value
Stage 1 (1–21 d)						
T-AOC, U/mg prot	68.20	70.00	73.24	71.08	1.37	0.517
SOD, U/mg prot	106.80 ^c^	134.22 ^b^	187.08 ^a^	179.73 ^a^	7.51	<0.001
GSH-Px, U/g prot	96.20 ^b^	97.27 ^b^	117.56 ^a^	123.69 ^a^	3.51	0.002
MDA, nmol/g prot	7.41	7.55	7.70	8.19	0.13	0.115
Whole-stage (1–42 d) ^1^						
T-AOC, U/mg prot	62.07 ^b^	76.08 ^a^	77.96 ^a^	73.42 ^a^	1.86	0.005
SOD, U/mg prot	120.80 ^c^	113.69 ^bc^	134.03 ^ab^	137.36 ^a^	3.08	0.015
GSH-Px, U/g prot	100.40 ^b^	94.31 ^b^	163.58 ^a^	107.24 ^b^	5.63	<0.001
MDA, nmol/g prot	5.73 ^b^	6.06 ^b^	6.12 ^b^	8.014 ^a^	0.20	<0.001

^1^ Whole-stage (1–42 d) values were calculated based on the complete 42-day dataset rather than by combining or averaging the two stage-specific values. ^a–c^ Values within a row with different superscripts differ significantly at *p* < 0.05. Abbreviations: T-AOC, total antioxidant capacity; SOD, superoxide dismutase; GSH-Px, glutathione peroxidase; MDA, malondialdehyde; SEM, pooled standard error of the mean.

**Table 6:** Effect of feed types on intestinal development of broilers (*n* = 8).

Items	CON	TG1	TG2	TG3	SEM	*p*-Value
Stage 1 (1–21 d)						
Duodenum, cm	11.98	12.31	12.07	13.45	0.28	0.461
Jejunum, cm	20.94 ^b^	24.27 ^a^	24.79 ^a^	23.91 ^a^	0.51	0.007
Ileum, cm	19.84 ^b^	23.03 ^b^	25.56 ^a^	26.02 ^a^	0.65	0.003
Cecum, cm	8.92 ^c^	9.07 ^bc^	9.88 ^ab^	10.01 ^a^	0.16	0.047
Whole-stage (1–42 d) ^1^						
Duodenum, cm	29.16	30.01	30.95	32.21	0.43	0.068
Jejunum, cm	70.58	72.25	72.8	72.57	0.47	0.345
Ileum, cm	70.10 ^b^	71.49 ^ab^	73.95 ^a^	73.72 ^a^	0.6	0.013
Cecum, cm	16.49	16.63	17.38	17.35	0.23	0.405

^1^ Whole-stage (1–42 d) values were calculated based on the complete 42-day dataset rather than by combining or averaging the two stage-specific values. ^a–c^ Values within a row with different superscripts differ significantly at *p* < 0.05.

**Table 7:** Effect of feed types on intestinal morphology of broilers (*n* = 8).

Items	CON	TG1	TG2	TG3	SEM	*p*-Value
Stage 1 (1–21 d)						
Duodenum						
Vh, μm	981.42 ^b^	1108.66 ^a^	1098.36 ^a^	1105.13 ^a^	15.74	<0.001
Cd, μm	218.83	214.34	210.38	210.06	1.43	0.11
V/C	4.49 ^b^	5.17 ^a^	5.21 ^a^	5.25 ^a^	0.08	<0.001
Jejunum						
Vh, μm	647.59 ^b^	808.28 ^a^	835.35 ^a^	823.86 ^a^	19.33	<0.001
Cd, μm	205.56	205.35	203.13	202.36	0.87	0.628
V/C	3.15 ^b^	3.93 ^a^	4.09 ^a^	4.06 ^a^	0.09	<0.001
Ileum						
Vh, μm	543.18 ^b^	647.09 ^a^	647.05 ^a^	642.6 ^a^	11.89	<0.001
Cd, μm	157.47	155.26	151.18	155.95	0.92	0.145
V/C	3.46 ^b^	4.17 ^a^	4.28 ^a^	4.12 ^a^	0.09	<0.001
Whole-stage (1–42 d) ^1^						
Duodenum						
Vh, μm	1133.68 ^c^	1258.52 ^b^	1356.24 ^a^	1299.91 ^ab^	18.42	<0.001
Cd, μm	241.77 ^a^	240.82 ^a^	232.87 ^b^	231.53 ^b^	1.32	0.003
V/C	4.69 ^d^	5.22 ^c^	5.82 ^a^	5.62 ^b^	0.08	<0.001
Jejunum						
Vh, μm	844.01 ^c^	991.56 ^b^	1032.25 ^a^	974.18 ^b^	13.85	<0.001
Cd, μm	226.12 ^a^	218.24 ^b^	220.07 ^b^	223.62 ^ab^	1.07	0.033
V/C	3.73 ^c^	4.55 ^a^	4.69 ^a^	4.36 ^b^	0.07	<0.001
Ileum						
Vh, μm	723.77 ^b^	823.67 ^a^	853.70 ^a^	836.72 ^a^	10.27	<0.001
Cd, μm	198.47	196.79	198.43	198.91	0.64	0.686
V/C	3.65 ^c^	4.19 ^b^	4.30 ^a^	4.21 ^ab^	0.05	<0.001

^1^ Whole-stage (1–42 d) values were calculated based on the complete 42-day dataset rather than by combining or averaging the two stage-specific values. ^a–d^ Values within a row with different superscripts differ significantly at *p* < 0.05. Abbreviations: Vh, villus height; Cd, crypt depth; V/C, ratio of villus height to crypt depth; SEM, pooled standard error of the mean.

**Table 8:** Effect of feed types on intestinal disaccharidase activity of broilers (*n* = 8).

Items	CON	TG1	TG2	TG3	SEM	*p*-Value
Stage 1 (1–21 d)						
Duodenum						
Maltase, U/mg	58.35	61.3	62.64	61.37	1.15	0.329
Sucrase, U/mg	28.30	29.32	32.08	29.91	1.20	0.579
Jejunum						
Maltase, U/mg	110.41 ^b^	121.77 ^a^	124.15 ^a^	123.95 ^a^	1.53	<0.001
Sucrase, U/mg	40.40 ^b^	57.86 ^a^	55.27 ^a^	54.51 ^a^	1.95	<0.001
Ileum						
Maltase, U/mg	85.01 ^b^	93.96 ^a^	97.91 ^a^	99.24 ^a^	1.42	<0.001
Sucrase, U/mg	38.10 ^b^	49.46 ^a^	46.84 ^a^	50.11 ^a^	1.45	<0.001
Whole-stage (1–42 d) ^1^						
Duodenum						
Maltase, U/mg	64.70 ^c^	75.66 ^b^	84.05 ^a^	71.39 ^b^	1.51	<0.001
Sucrase, U/mg	29.18 ^b^	35.82 ^a^	36.17 ^a^	32.41 ^ab^	0.82	0.003
Jejunum						
Maltase, U/mg	122.51 ^d^	148.96 ^c^	175.29 ^a^	164.21 ^b^	3.78	<0.001
Sucrase, U/mg	67.61 ^c^	75.45 ^b^	86.02 ^a^	83.22 ^a^	1.54	<0.001
Ileum						
Maltase, U/mg	95.54 ^b^	100.43 ^a^	103.03 ^a^	101.18 ^a^	0.68	<0.001
Sucrase, U/mg	39.27 ^b^	45.44 ^a^	49.66 ^a^	46.48 ^a^	1.07	0.002

^1^ Whole-stage (1–42 d) values were calculated based on the complete 42-day dataset rather than by combining or averaging the two stage-specific values. ^a–d^ Values within a row with different superscripts differ significantly at *p* < 0.05. Abbreviations: SEM, pooled standard error of the mean.

**Table 9:** Effect of feed types on α-diversity in cecum microbiota of broilers.

Items	CON	TG1	TG2	TG3	SEM	*p*-Value
Whole-stage (1–42 d)						
Ace	494.83 ^a^	479.27 ^ab^	477.80 ^ab^	449.55 ^b^	6.32	0.025
Chao1	501.45 ^a^	483.11 ^ab^	483.37 ^ab^	454.21 ^b^	6.82	0.031
Shannon	3.90	3.97	4.12	4.16	0.50	0.539
Simpson	0.07 ^a^	0.07 ^a^	0.05 ^ab^	0.04 ^b^	0.01	0.014

Data represent the means of 8 broiler chickens per group. ^a,b^ Values within a row with different superscripts differ significantly at *p* < 0.05.

## Data Availability

Data will be made available on request.

## Supplementary Materials

The following supporting information can be downloaded at: https://www.mdpi.com/article/10.3390/ani16152308/s1, S1: European patent on SPF; Table S1: Analysis of microbial α diversity in Stage 1 broilers; Figure S1: Analysis of the significant differences in the correlation between cecal microbiota and growth performance, intestinal development, liver and kidney health, and antioxidant capacity after the end of stage 1; Figure S2: Analysis of the significant differences in the correlation between cecal microbiota and growth performance, intestinal development, liver and kidney health, and antioxidant capacity after the end of stage 2.

## Abbreviations

The following abbreviations are used in this manuscript:

ADFI	Average Daily Feed Intake
ADG	Average Daily Gain
ALT	Alanine Aminotransferase
AST	Aspartate Aminotransferase
CPF	Crumbled-Pellet Feed
CREA	Creatinine
DMFCR	Dry Matter Feed Conversion Ratio
FBW	Final Body Weight
FCR	Feed Conversion Ratio
Glu	Glucose
GSH-Px	Glutathione Peroxidase
H&E	Hematoxylin–Eosin
HPF	Hard-Pelleted Feed
MDA	Malondialdehyde
OTU	Operational Taxonomic Unit
SOD	Superoxide Dismutase
SPF	Soft-Pellet Feed
TC	Total Cholesterol
TGs	Triglycerides
T-AOC	Total Antioxidant Capacity
UA	Uric Acid
