# Supplementary material for: Soft-Pellet Feed Strategy for Broilers: Effects on Growth Performance and Liver–Intestinal Health
Source: Animals (Basel). 2026 Jul 26;16(15):2308. doi: 10.3390/ani16152308 (PMC13463713; doi:10.3390/ani16152308)
Supplement: Supplementary file 1 [file animals-16-02308-s001.zip › Supplementary Material S1.pdf]

(19) Europäisches Patentamt
European Patent Office
Office européen des brevets

(11) EP 3 251 523 A1

(12) EUROPEAN PATENT APPLICATION

(43) Date of publication:
06.12.2017 Bulletin 2017/49

(21) Application number: 17000914.6

(22) Date of filing: 30.05.2017

(51) Int Cl.:
A23K 50/60 (2016.01) *A23K 40/25* (2016.01)
A23K 50/30 (2016.01)

(84) Designated Contracting States:
AL AT BE BG CH CY CZ DE DK EE ES FI FR GB GR HR HU IE IS IT LI LT LU LV MC MK MT NL NO PL PT RO RS SE SI SK SM TR
Designated Extension States:
BA ME
Designated Validation States:
MA MD

(30) Priority: 31.05.2016 CN 201610375166

(71) Applicant: Wuhan Polytechnic University
Wuhan, Hubei 430023 (CN)

(72) Inventors:
- WANG, Chunwei
Wuhei, Hubei, 430023 (CN)
- WU, Fengwei
Wuhei, Hubei, 430023 (CN)
- ZHANG, Aixia
Wuhei, Hubei, 430023 (CN)
- HAN, Pengwei
Wuhei, Hubei, 430023 (CN)
- BU, Xiaoli
Wuhei, Hubei, 430023 (CN)
- LIU, Shicao
Wuhei, Hubei, 430023 (CN)
- CHEN, Fan
Wuhei, Hubei, 430023 (CN)

(74) Representative: Zeuner Summerer Stütz
Nußbaumstrasse 8
80336 München (DE)

(54) METHOD FOR PREPARING GRANULATED CREEP FEED

(57) A method for preparing granulated creep feed, which solves the problem of being not palatable for piglets to digest and having a short storage time. The method including: 1) uniformly mixing 100 parts by weight of a powder material with between 24 and 30 parts by weight of water to yield a mixture; 2)heating the mixture at a first heating stage to a first temperature of 45-60°C, and maintaining the first temperature for 25-30 s; heating the mixture at a second heating stage to a second temperature of 65-90°C, and maintaining the second temperature for 20-25 s; and heating the mixture at a third heating stage to a third temperature of 95-120°C, and maintaining the third temperature for 15-20 s to yield an aged material; and 3) extruding the aged material into strips by an extruding device, cutting the strips into granules, and dispersing and cooling the granules. The method of the invention is defined by short production cycle and low investment, production, and storage costs. The prepared granules are of low hardness, are palatable for piglets, and are easy to digest.

Printed by Jouve, 75001 PARIS (FR)

Description

FIELD OF THE INVENTION

[0001] The invention relates to a filed of feed, specially relates to a method for preparing granulated creep feed.

BACKGROUND OF THE INVENTION

[0002] In modem pig husbandry, in order to improve production efficiency, shorten the lactation of piglets and improve the breeding efficiency of sows, the days for weaning is reduced to 21 to 28 days from the traditional 45 to 50 days and the annual output of the original breeding sows is increased to 2.4 to 2.6 nests from an annual output of below 2 nests. The technology is called early weaning technology of piglets. Early use of weaning technology has greatly improved the utilization of pig facilities, the production efficiency of sows and the labor productivity. The sow feeding is reduced by 15% (sows do not bring direct benefits) by the early weaning technology. Although the early weaning technology has effectively improved the production efficiency of the sows, it poses a serious challenge to the early weaning piglets that the piglets have a series of changes in complex nutritional, physiological and behavioral during the weaning process. Thus, the piglets should overcome the piglets weaning syndromes, such as the emergence of diarrhea, reduced feed intake, growth retardation, decreased immunity, increased mortality etc., affected by stress factors, such as nutrition, psychology, environment etc., which are caused by the early weaning.

[0003] For a long time, the feed preparation and the breeding technology of the early weaning piglets are hotpot and difficult point in the domestic and foreign animal nutrition research. Feeding habits of piglets before and after weaning are called creep feed. The important indicators of growth performance of the piglets before and after weaning which are used to evaluate the creep feed are the feed intake and diarrhea rate. At present, there are three types of granulated feed, mash feed and liquid feed. The granulated feed and mash feed are morphologically different from breast milk of sows, which adversely affects the daily intake amount of feed and the normal growth of the piglets. Although the liquid feed is closer to the breast milk of animals, the liquid feed often has low nutrient concentration and cannot satisfy the requirements for the fast growth of the piglets. Thus, it is need to develop a transition production between the liquid feed and the granulated feed. On one hand, it hopes that the process can be suitable for various creep feed materials without specially improving the ingredients of the raw materials. On the other hand, it hopes that the preparation process is as simple as possible and the production cost is reduced.

[0004] In addition, conventional creep feed has a relatively short storage time and requires stringent storage conditions, leading to high transportation and storage costs.

SUMMARY OF THE INVENTION

[0005] In view of the above-described problems, it is one objective of the invention to provide a method for preparing granulated creep feed that is defined by short production cycle and low investment, production, and storage costs. The prepared granules are of low hardness, are palatable for piglets, and are easy to digest.

[0006] The method comprises:

1) uniformly mixing 100 parts by weight of a powder material with between 24 and 30 parts by weight of water to yield a mixture;

2) heating the mixture obtained from step 1) in three consecutive stages, heating the mixture at a first heating stage to a first temperature of between 45 and 60°C, and maintaining the first temperature for between 25 and 30 s; heating the mixture at a second heating stage to a second temperature of between 65 and 90°C, and maintaining the second temperature for between 20 and 25 s; and heating the mixture at a third heating stage to a third temperature of between 95 and 120°C, and maintaining the third temperature for between 15 and 20 s to yield an aged material; and

3) extruding the aged material into strips by an extruding device, cutting the strips into granules, and dispersing and cooling the granules to yield an aged granulated creep feed.

[0007] In a class of this embodiment, between 0.2 and 0.5 part by weight of a compound acidifier is added in step 1); the compound acidifier comprises: between 50.0 and 70.0 parts by weight of sodium diacetate, between 10.0 and 28.0 parts by weight of sodium benzoate, between 10.0 and 30.0 parts by weight of potassium sorbate, between 0.5 and 1.0 part by weight of nisin, and between 4.0 and 10.0 parts by weight of citric acid.

[0008] In a class of this embodiment, in step 3), a starch retrogradation inhibitor and a humectant are dissolved into water to yield a solution; the solution is sprayed to surfaces of dispersed and cooled granules by using vacuum spraying,

and the granules are air dried to yield the aged creep feed in the form of the granules.

[0009] In a class of this embodiment, the starch retrogradation inhibitor comprises: α-amylase and β-amylase; the humectant is D-sorbitol; an addition of the starch retrogradation inhibitor is 0.5 part by weight, and an addition of the humectant is 1 part by weight.

[0010] In a class of this embodiment, in the first heating stage, the mixture is heated to between 55 and 60°C; in the second heating stage, the mixture is heated to between 85 and 90°C; and in the third heating stage, the mixture is heated to between 115 and 120°C.

[0011] In a class of this embodiment, a particle size of the aged granulated creep feed is 5 mm.

[0012] In a class of this embodiment, in 1), between 0.2 and 0.5 part by weight of the compound acidifier is dissolved in between 24 and 30 parts by weight of the water at a temperature of 60°C to yield a solution, then 100 parts by weight of the powder material is added and uniformly mixed with the solution.

[0013] The feeds in the prior art have the following problems: the feed in the form of hard granules has relatively large hardness which is difficult for the administration of the piglets; the powder feed is easily to be fed but it has poor palatability, results in great waste and inflammation as the powder feed is apt to adhere to the respiratory passage; the wet stirred material is difficult to be stored and the transportation cost is high. The invention is aimed to provide an aged creep feed in the form of soft granules that has good palatability and easily digestion, and is suitable for long-period storage.

[0014] The mixture of the powder material and the water is heated in three consecutive stages: the mixture is firstly heated to between 45 and 60°C, followed with a temperature preservation of between 25 and 30 s, so as to preliminarily age the creep feed; thereafter, the mixture is heated to between 65 and 90°C, followed with a temperature preservation of between 20 and 25 s, so as to be fully conditioned; and finally, the mixture is heated to between 95 and 120°C, followed with a temperature preservation of between 15 and 20 s, so as to further age the mixture. During the three-heating stages, the preservation times after each heating stage should be controlled. Too long the heating stage easily results in starch retrogradation, and too short the heating stage leads uneven aging of the mixture. By the three-stage heating, the over-gelatinization of the starch is avoided. The three-stage heating is able to effectively improve the gelatinization degree of the starch to be equal to or larger than 43%; accordingly, the production efficiency of the method is high, the granules are soft and not prone to agglomerate into masses, the nutrient gradients are well stored, and the palatability is good. As the simple heating process are able to prepare the soft granules, compared with other complicate processes for preparation of soft granules, the production process of the invention is much shorter, the operation control is much simpler, and the production cost is much lower, thus being suitable to prepare different kinds of creep feeds. In the method of the invention, the three heating stages and the preservation processes of the mixtures including the powder material can be performed in a sleeve of the extruding device, and it only requires to control the temperatures of the three stages and the transporting speed of the materials in each stage.

[0015] Furthermore, to improve the storage property of the aged granulated creep feed, the following improvements of the invention are made:

1. Compound acidifier is added to the powered material. The compound acidifier has the following advantages: a. the compound acidifier is able to improve the acid environment of the intestine of the piglets and the digestibility of the feeder accordingly; b. The compound acidifier possesses relatively strong sterilizing and broad-spectrum anti-bacterial properties; c. the compound acidifier is able to improve the palatability, the intake amount, and the digestibility of the feed. The components of the compound acidifier are all food materials, in which, sodium diacetate and potassium sorbate are coordinate for mildewproof. The mildewproof property of a combination of sodium diacetate and potassium sorbate is significantly improved and is 6.3 folds higher than the single use of sodium diacetate. In addition, the compound acidifier of the invention further includes sodium benzoate, which is anti-bacterial. The addition of Nisin is aimed to provide amino acids at the same time of anti-bacteria. And the mildewproof property of the combination of components is 1.5 folds higher than the combined use of sodium diacetate and potassium sorbate. Furthermore, in the mixed phase in 1), water at 60°C is mixed with the compound acidifier and the powered material, which makes the starch preliminarily gelatinized. The gelatinization of the feed is further improved in subsequent processing steps to make the physical and chemical properties of the granules of the product much better.

2. The granules after extruding and cutting are further processed by vacuum spraying to spray the starch retrogradation inhibitor and the humectant and form a coating on the surface of the granule. Not only the starch retrogradation is effectively inhibited, but also the moisturizing, fresh-keeping, and anti-bacterial effects are ensured. The coating is equivalent to an "outerwear" provided on the surface of the particulars, so that the proliferation of mould is inhibited and the expiration date is prolonged.

[0016] The powered material of the invention is not limited especially. The powered material of the invention can be the normally used powered material for the creep feed in the prior art. The starch retrogradation inhibitor preferably comprises: α-amylase and β-amylase. The addition of the starch retrogradation inhibitor is preferably 0.2 part by weight,

and the addition of the humectant is preferably 0.3 part by weight. The humectant is preferably D-sorbitol.

[0017] Advantages of the method for preparing the aged granulated creep feed according to embodiments of the invention are summarized as follows:

[0018] The method of the invention adopts multiple-stage mildewproof means and modem food and feed processing techniques to effectively improve the mildewproof, water-keeping, moisturizing, and anti-retrogradation properties. A daily intake amount of the aged granulated creep feed prepared by the method of the invention is 1.68 folds of those of the feeds prepared by the conventional methods. The hardness of the granules of the feed is significantly reduced, which is reduced by 71.7% and 43.9% respectively compared with the conventional low-temperature hard granule creep feed and the crushed granule creep feed. The aged granulated creep feed of the invention is able to effectively improve the digestion and alleviate weaning stress of the piglets, and reduces the diarrhea rate by 59.2%. The aging degree (gelatinization degree) of the creep feed is greatly improved by the heating condition and reaches 61%, which is two times of the conventional creep feed. It is indicated that the growth of the piglet fed with the aged granulated creep feed of the invention is significantly better than those fed with the non-aged hard granules of feed, and the daily weight gain of the piglets fed with the creep feed of the invention one week after the weaning is increased by 49.5%. The prepared aged granulated creep feed is able to be preserved for 40 days at an environment temperature of 35°C, and for 48 days at the environment temperature of 25°C, thus the preservation period is greatly improved. The production process is simple, easily controlled, the production cost is low, and no dust or effluent pollution is resulted. The creep feed prepared has relatively high gelatinization degree, high digestibility, excellent palatability, and extensive market prospect.

DETAILED DESCRIPTION OF THE EMBODIMENTS

[0019] The following percentage is weight percentage.

[0020] A powder material comprises: 54 parts by weight of extruded corns, 15 parts by weight of extruded soybeans, 4 parts by weight of steamed fish powder, 6 parts by weight of a fermented soybean meal, 4 parts by weight of a spray-dried plasma protein, 4 parts by weight of a glucose, 5 parts by weight of a milk powder, 4.5 parts by weight of an egg powder, 0.4 part by weight of lysine, 0.3 part by weight of methionine, 0.1 part by weight of threonine, 0.05 part by weight of tryptophan, 2.5 parts by weight of a compound-premix, a flavoring agent including 0.05 part by weight of a ground cinnamon and 0.1 part by weight of betaine.

Example 1

[0021]

1) 0.2 part by weight of the compound acidifier was added to 24 parts by weight of water at a temperature of 60°C and uniformly mixed. Thereafter, 100 parts by weight of a powder material was added, and a resulting mixture was uniformly stirred in a dough kneader for 10 min to avoid spherical granules.

2) Materials obtained from step 1) were heated for three heating stages while stirring. The materials were firstly heated to 60°C, followed by temperature preservation of between 25 and 30 s; then heated to 90°C, followed by temperature preservation of between 20 and 25 s; and finally heated to 120°C, followed by temperature preservation of between 15 and 20 s to yield aged materials.

3)The aged materials were extruded into strips by an extruder, and cut into granules having a particle size of 2 mm. The granules were dispensed and cooled. 0.03 part by weight of α-amylase, 0.2 part by weight of β-amylase, and 1 part by weight of a humectant (D-sorbitol) were mixed to prepare an aqueous solution. The aqueous solution was then sprayed onto surfaces of the granules of the aged materials. The granules of the aged materials were air dried to obtain aged granules of a creep feed.

[0022] The aged granulated creep feed was packed by a sterilized uniform membrane bag. The aged granulated creep feed prepared in this example was labeled as sample 1.

Example 2

[0023]

1) 0.3 part by weight of the compound acidifier was added to 24 parts by weight of water at a temperature of 60°C and uniformly mixed. Thereafter, 100 parts by weight of a powder material was added, and a resulting mixture was uniformly stirred in a dough kneader for 10 min to avoid spherical granules.

2) Materials obtained from 1) were heated for three heating stages while stirring. The materials were firstly heated to 60°C, followed by temperature preservation of between 25 and 30 s; then heated to 90°C, followed by temperature preservation of between 20 and 25 s; and finally heated to 120°C, followed by temperature preservation of between 15 and 20 s to yield aged materials.

3)The aged materials were extruded into strips by an extruder, and cut into granules having a particle size of 2 mm. The granules were dispensed and cooled. 0.03 part by weight of α-amylase, 0.2 part by weight of β-amylase, and 1 part by weight of a humectant (D-sorbitol) were mixed to prepare an aqueous solution. The aqueous solution was then sprayed onto surfaces of the granules of the aged materials. The granules of the aged materials were air dried to obtain aged granules of a creep feed.

[0024] The aged granulated creep feed was packed by a sterilized uniform membrane bag. The aged granulated creep feed prepared in this example was labeled as sample 2.

Example 3

[0025]

1) 0.3 part by weight of the compound acidifier was added to 26 parts by weight of water at a temperature of 60°C and uniformly mixed. Thereafter, 100 parts by weight of a powder material was added, and a resulting mixture was uniformly stirred in a dough kneader for 10 min to avoid spherical granules.

2) Materials obtained from step 1) were heated for three heating stages while stirring. The materials were firstly heated to 45°C, followed by temperature preservation of between 25 and 30 s; then heated to 65°C, followed by temperature preservation of between 20 and 25 s; and finally heated to 95°C, followed by temperature preservation of between 15 and 20 s to yield aged materials.

3)The aged materials were extruded into strips by an extruder, and cut into granules having a particle size of 2 mm. The granules were dispensed and cooled. 0.03 part by weight of α-amylase, 0.2 part by weight of β-amylase, and 1 part by weight of a humectant (D-sorbitol) were mixed to prepare an aqueous solution. The aqueous solution was then sprayed onto surfaces of the granules of the aged materials. The granules of the aged materials were air dried to obtain aged granules of a creep feed.

[0026] The aged granulated creep feed was packed by a sterilized uniform membrane bag. The aged granulated creep feed prepared in this example was labeled as sample 3.

Example 4

[0027]

1) 0.3 part by weight of the compound acidifier was added to 26 parts by weight of water at a temperature of 60°C and uniformly mixed. Thereafter, 100 parts by weight of a powder material was added, and a resulting mixture was uniformly stirred in a dough kneader for 10 min to avoid spherical granules.

2) Materials obtained from step 1) were heated for three heating stages while stirring. The materials were firstly heated to 60°C, followed by temperature preservation of between 25 and 30 s; then heated to 90°C, followed by temperature preservation of between 20 and 25 s; and finally heated to 120°C, followed by temperature preservation of between 15 and 20 s to yield aged materials.

3)The aged materials were extruded into strips by an extruder, and cut into granules having a particle size of 2 mm. The granules were dispensed and cooled. 0.03 part by weight of α-amylase, 0.2 part by weight of β-amylase, and 1 part by weight of a humectant (D-sorbitol) were mixed to prepare an aqueous solution. The aqueous solution was then sprayed onto surfaces of the granules of the aged materials. The granules of the aged materials were air dried to obtain aged granules of a creep feed.

[0028] The aged granulated creep feed was packed by a sterilized uniform membrane bag. The aged granulated creep feed prepared in this example was labeled as sample 4.

Example 5

[0029]

1) 0.4 part by weight of the compound acidifier was added to 26 parts by weight of water at a temperature of 60°C and uniformly mixed. Thereafter, 100 parts by weight of a powder material was added, and a resulting mixture was uniformly stirred in a dough kneader for 10 min to avoid spherical granules.

2) Materials obtained from 1) were heated for three heating stages while stirring. The materials were firstly heated to 60°C, followed by temperature preservation of between 25 and 30 s; then heated to 90°C, followed by temperature preservation of between 20 and 25 s; and finally heated to 120°C, followed by temperature preservation of between 15 and 20 s to yield aged materials.

3)The aged materials were extruded into strips by an extruder, and cut into granules having a particle size of 2 mm. The granules were dispensed and cooled. 0.03 part by weight of α-amylase, 0.2 part by weight of β-amylase, and 1 part by weight of a humectant (D-sorbitol) were mixed to prepare an aqueous solution. The aqueous solution was then sprayed onto surfaces of the granules of the aged materials. The granules of the aged materials were air dried to obtain aged granules of a creep feed.

[0030] The aged granulated creep feed was packed by a sterilized uniform membrane bag. The aged granulated creep feed prepared in this example was labeled as sample 5.

Example 6

[0031]

1) 0.4 part by weight of the compound acidifier was added to 26 parts by weight of water at a temperature of 60°C and uniformly mixed. Thereafter, 100 parts by weight of a powder material was added, and a resulting mixture was uniformly stirred in a dough kneader for 10 min to avoid spherical granules.

2) Materials obtained from 1) were heated for three heating stages while stirring. The materials were firstly heated to 55°C, followed by temperature preservation of between 25 and 30 s; then heated to 85°C, followed by temperature preservation of between 20 and 25 s; and finally heated to 110°C, followed by temperature preservation of between 15 and 20 s to yield aged materials.

3)The aged materials were extruded into strips by an extruder, and cut into granules having a particle size of 2 mm. The granules were dispensed and cooled. 0.03 part by weight of α-amylase, 0.2 part by weight of β-amylase, and 1 part by weight of a humectant (D-sorbitol) were mixed to prepare an aqueous solution. The aqueous solution was then sprayed onto surfaces of the granules of the aged materials. The granules of the aged materials were air dried to obtain aged granules of a creep feed.

[0032] The aged granulated creep feed was packed by a sterilized uniform membrane bag. The aged granulated creep feed prepared in this example was labeled as sample 6.

Example 7

[0033]

1) 0.3 part by weight of the compound acidifier was added to 28 parts by weight of water at a temperature of 60°C and uniformly mixed. Thereafter, 100 parts by weight of a powder material was added, and a resulting mixture was uniformly stirred in a dough kneader for 10 min to avoid spherical granules.

2) Materials obtained from 1) were heated for three heating stages while stirring. The materials were firstly heated to 60°C, followed by temperature preservation of between 25 and 30 s; then heated to 90°C, followed by temperature preservation of between 20 and 25 s; and finally heated to 120°C, followed by temperature preservation of between 15 and 20 s to yield aged materials.

3)The aged materials were extruded into strips by an extruder, and cut into granules having a particle size of 2 mm.

The granules were dispensed and cooled. 0.03 part by weight of α-amylase, 0.2 part by weight of β-amylase, and 1 part by weight of a humectant (D-sorbitol) were mixed to prepare an aqueous solution. The aqueous solution was then sprayed onto surfaces of the granules of the aged materials. The granules of the aged materials were air dried to obtain aged granules of a creep feed.

[0034] The aged granulated creep feed was packed by a sterilized uniform membrane bag. The aged granulated creep feed prepared in this example was labeled as sample 7.

Example 8

[0035]

1) 0.4 part by weight of the compound acidifier was added to 28 parts by weight of water at a temperature of 60°C and uniformly mixed. Thereafter, 100 parts by weight of a powder material was added, and a resulting mixture was uniformly stirred in a dough kneader for 10 min to avoid spherical granules.

2) Materials obtained from 1) were heated for three heating stages while stirring. The materials were firstly heated to 60°C, followed by temperature preservation of between 25 and 30 s; then heated to 90°C, followed by temperature preservation of between 20 and 25 s; and finally heated to 120°C, followed by temperature preservation of between 15 and 20 s to yield aged materials.

3)The aged materials were extruded into strips by an extruder, and cut into granules having a particle size of 2 mm. The granules were dispensed and cooled. 0.03 part by weight of α-amylase, 0.2 part by weight of β-amylase, and 1 part by weight of a humectant (D-sorbitol) were mixed to prepare an aqueous solution. The aqueous solution was then sprayed onto surfaces of the granules of the aged materials. The granules of the aged materials were air dried to obtain aged granules of a creep feed.

[0036] The aged granulated creep feed was packed by a sterilized uniform membrane bag. The aged granulated creep feed prepared in this example was labeled as sample 8.

Example 9

[0037]

1) 0.4 part by weight of the compound acidifier was added to 26 parts by weight of water at a temperature of 60°C and uniformly mixed. Thereafter, 100 parts by weight of a powder material was added, and a resulting mixture was uniformly stirred in a dough kneader for 10 min to avoid spherical granules.

2) Materials obtained from 1) were heated for three heating stages while stirring. The materials were firstly heated to 60°C, followed by temperature preservation of between 25 and 30 s; then heated to 90°C, followed by temperature preservation of between 20 and 25 s; and finally heated to 120°C, followed by temperature preservation of between 15 and 20 s to yield aged materials.

3)The aged materials were extruded into strips by an extruder, and cut into granules having a particle size of 2 mm. The granules were dispensed and cooled. 0.03 part by weight of α-amylase, 0.2 part by weight of β-amylase, and 1 part by weight of a humectant (D-sorbitol) were mixed to prepare an aqueous solution. The aqueous solution was then sprayed onto surfaces of the granules of the aged materials. The granules of the aged materials were air dried to obtain aged granules of a creep feed.

[0038] The aged granulated creep feed was packed by a sterilized uniform membrane bag. The aged granulated creep feed prepared in this example was labeled as sample 9.

Example 10

[0039]

1) 0.4 part by weight of the compound acidifier was added to 30 parts by weight of water at a temperature of 60°C and uniformly mixed. Thereafter, 100 parts by weight of a powder material was added, and a resulting mixture was

uniformly stirred in a dough kneader for 10 min to avoid spherical granules.

2) Materials obtained from 1) were heated for three heating stages while stirring. The materials were firstly heated to 60°C, followed by temperature preservation of between 25 and 30 s; then heated to 90°C, followed by temperature preservation of between 20 and 25 s; and finally heated to 120°C, followed by temperature preservation of between 15 and 20 s to yield aged materials.

3)The aged materials were extruded into strips by an extruder, and cut into granules having a particle size of 2 mm. The granules were dispensed and cooled. 0.03 part by weight of α-amylase, 0.2 part by weight of β-amylase, and 1 part by weight of a humectant (D-sorbitol) were mixed to prepare an aqueous solution. The aqueous solution was then sprayed onto surfaces of the granules of the aged materials. The granules of the aged materials were air dried to obtain aged granules of a creep feed.

[0040] The aged granulated creep feed was packed by a sterilized uniform membrane bag. The aged granulated creep feed prepared in this example was labeled as sample 10.

Comparative example 1

[0041] The powder material comprising the above ingredients were directly extruded by the extruding machine to yield a feed in the form of hard granules.

[0042] Physical and chemical properties of products and growth indicators of animals

1) Physical analysis

[0043]

Table 1 Detection results of physical and chemical properties of aged granulated creep feed.

Sample	Flavor	Appearance	Gelatinization degree/%	Storage period/day	
				At 25°C	At 35°C
Sample 1	Milk flavor	Yellow	56.8	40	37
Sample 2	Slight acid flavor	Yellow	56.7	40	38
Sample 3	Milk flavor	Yellow	43.2	39	37
Sample 4	Milk flavor	Brown	59.3	37	32
Sample 5	Slight acid flavor	Brown	60.1	48	41
Sample 6	Slight acid flavor	Brown	48.8	35	28
Sample 7	Milk flavor	Dark brown	60.4	31	26
Sample 8	Slight acid flavor	Dark brown	60.8	34	29
Sample 9	Slight acid flavor	Brown	56.5	28	25
Sample 10	Slight acid flavor	Brown	56.1	26	24

2) Feeding experiments

A. Experiment conditions

[0044]

Experiment period: between Jan. 8, 2016 and Jan. 29, 2016

Weaning time: Jan. 21, 2016

Experiment site: an experimental pig breeder site in Hubei Province

Temperature:

Cold wave period: between Jan. 20, 2016 and Jan. 24, 2016

Outdoor: between -7 and 10°C

Indoor: between 9 and 14°C

B. Experiment

[0045] Groups: four groups with each group including four repeats were designed. Each brood of each repeat including between 7 and 11 piglets (a total of 146 piglets). The piglets of all repeats were arranged according to an ascending order of weight, and then divided into four groups (A, B, C, and D) according to an S-type division.

Group A: piglets were fed with the feed in the form of hard granules in Comparative example 1;

Group B: piglets were fed with a powder feed.

Group C: piglets were fed with a commercially available feed in the form of hard granules; and

Group D: piglets were fed with the aged creep feed in the form soft granules obtained from Example 5.

3. Main growth indicators

[0046]

Table 2 Daily intake amount (g)

Growing periods	Groups			
	A (materials of hard granules)	B (powder material)	C (materials of hard granules)	D (materials of soft granules)
7-21 days old (before weaning)	14.07	15.92	12.07	20.74 (17.6)
One week after weaning	137.90	197.87	157.08	231.89(196)

Table 3 Average weight of piglet (kg)

Growth stage	Groups			
	Group A	Group B	Group C	Group D
7-day old	2.8250	2.8481	2.8385	2.8366
21-day old	5.4639	5.6302	5.2331	6.4914
One week after weaning	6.2159	6.5188	5.9322	7.6139

Table 4 Daily weight gain (g)

Growth stage	Groups			
	Group A	Group B	Group C	Group D
7-21-day old (before weaning)	188.49	198.72	171.04	261.05
One week after weaning	107.43	126.94	99.88	160.36

Table 5 Ratio of feed material to weight gain

Growth stage	Groups			
	Group A	Group B	Group C	Group D
7-21-day old (before weaning)	0.07463	0.08010	0.07054	0.07946
One week after weaning	1.2837	1.5587	1.5728	1.4460(1.23)

Table 6 Fur condition (A Very coarse; B Coarse; C. Relatively smooth; D. Smooth; and E. Very smooth)

Growth stage	Groups			
	Group A	Group B	Group C	Group D
0-7-day old	D	D	D	D
7-21-day old	D	D	D	D
One week after weaning	C	D	D	C
Whole period	C	D	D	C

Table 7 Diarrhea rate (%)

Growth stage	Groups			
	Group A	Group B	Group C	Group D
7-21-day old (before weaning)	0.45	0.84	0.65	0
One week after weaning	1.79	2.94	1.30	0.73

Conclusions

[0047]

1) Mortality (%): during the whole feeding period, no death happened in each group.

2) It was known from the process from creep feed attracting of the 7-day old piglets, weaning of the 21-day old piglets, and creep feed feeding of piglets one week after the weaning that: a. the daily intake amount of the group D was significantly higher than other three groups during the whole process, and was 1.68 folds of the group A, indicating that the aged creep feed in the form of the soft granules has the best palatability and relatively strong feed attracting effect; b. the diarrhea rate of the group D was significantly lower than other three groups, and was reduced by 59.2% compared with the group A, indicating that the creep feed of group D was much better digested by the piglets; d. the aging degree (gelatinization degree of the starch) of the creep feed was greatly improved by the heating condition and reaches 61%, which was increased by 1 fold; and e. the growth of the piglets fed with the aged creep feed in the form of the soft granules is significantly higher than other groups, and the daily weight gain of such group one week after the weaning is increased by 49.5% compared with the group A.

Claims

1. A method for preparing granulated creep feed, the method comprising:

1) uniformly mixing 100 parts by weight of a powder material with between 24 and 30 parts by weight of water to yield a mixture;
2) heating the mixture in three consecutive stages (i-iii):

(i) heating the mixture at a first heating stage to a first temperature of between 45 and 60°C, and maintaining the first temperature for between 25 and 30 s;
(ii) heating the mixture at a second heating stage to a second temperature of between 65 and 90°C, and maintaining the second temperature for between 20 and 25 s; and
(iii) heating the mixture at a third heating stage to a third temperature of between 95 and 120°C, and maintaining the third temperature for between 15 and 20 s, to yield an aged material; and

3) extruding the aged material into strips by an extruding device, cutting the strips into granules, and dispersing and cooling the granules to yield granulated creep feed.

2. The method of claim 1, wherein between 0.2 and 0.5 part by weight of a compound acidifier is added in step 1); the compound acidifier comprises: between 50.0 and 70.0 parts by weight of sodium diacetate, between 10.0 and 28.0

parts by weight of sodium benzoate, between 10.0 and 30.0 parts by weight of potassium sorbate, between 0.5 and 1.0 part by weight of nisin, and between 4.0 and 10.0 parts by weight of citric acid.

3. The method of claim 1, wherein in step 3), a starch retrogradation inhibitor and a humectant are dissolved into water to yield a solution; following dispersing and cooling of the granules, the solution is sprayed to surfaces of the granules by using vacuum spraying, and then the granules are air dried to yield an aged granulated creep feed.

4. The method of claim 3, wherein the starch retrogradation inhibitor comprises: α-amylase and β-amylase; the humectant is D-sorbitol; an addition of the starch retrogradation inhibitor is 0.5 part by weight, and an addition of the humectant is 1 part by weight.

5. The method of claim 1, wherein in the first heating stage, the mixture is heated to between 55 and 60°C; in the second heating stage, the mixture is heated to between 85 and 90°C; and in the third heating stage, the mixture is heated to between 115 and 120°C.

6. The method of any one of claims 1-6, wherein a particle size of the granulated creep feed is 5 mm.

7. The method of claim 2, wherein in sep 1), between 0.2 and 0.5 part by weight of the compound acidifier is dissolved in between 24 and 30 parts by weight of the water at a temperature of 60°C to yield a solution, then 100 parts by weight of the powder material is added and uniformly mixed with the solution.

Europäisches Patentamt
European Patent Office
Office européen des brevets

EUROPEAN SEARCH REPORT

Application Number
EP 17 00 0914

DOCUMENTS CONSIDERED TO BE RELEVANT

Category	Citation of document with indication, where appropriate, of relevant passages	Relevant to claim	CLASSIFICATION OF THE APPLICATION (IPC)
A	SUN T ET AL: "The effect of extrusion cooking of different starch sources on the in vitro and in vivo digestibility in growing pigs", ANIMAL FEED SCIENCE AND TECHNOLOGY, ELSEVIER, AMSTERDAM, NL, vol. 131, no. 1-2, 15 November 2006 (2006-11-15), pages 67-86, XP024934092, ISSN: 0377-8401, DOI: 10.1016/J.ANIFEEDSCI.2006.02.009 [retrieved on 2006-11-15] * the whole document * -----	1-7	INV. A23K50/60 A23K40/25 A23K50/30
A	L.N ZARKADAS ET AL: "Influence of micronization temperature and pre-conditioning on performance and digestibility in piglets fed barley-based diets", ANIMAL FEED SCIENCE AND TECHNOLOGY, vol. 95, no. 1-2, 1 January 2002 (2002-01-01), pages 73-82, XP055408629, AMSTERDAM, NL ISSN: 0377-8401, DOI: 10.1016/S0377-8401(01)00295-4 * the whole document * -----	1-7	TECHNICAL FIELDS SEARCHED (IPC): A23K

-/--

The present search report has been drawn up for all claims

Place of search	Date of completion of the search	Examiner
The Hague	25 September 2017	Heirbaut, Marc

CATEGORY OF CITED DOCUMENTS

X : particularly relevant if taken alone
Y : particularly relevant if combined with another document of the same category
A : technological background
O : non-written disclosure
P : intermediate document

T : theory or principle underlying the invention
E : earlier patent document, but published on, or after the filing date
D : document cited in the application
L : document cited for other reasons
& : member of the same patent family, corresponding document

4

EPO FORM 1503 03.82 (P04C01)

Europäisches Patentamt
European Patent Office
Office européen des brevets

EUROPEAN SEARCH REPORT

Application Number
EP 17 00 0914

DOCUMENTS CONSIDERED TO BE RELEVANT

Category	Citation of document with indication, where appropriate, of relevant passages	Relevant to claim	CLASSIFICATION OF THE APPLICATION (IPC)
A	K Hongtrakul ET AL: "The Effects of Extrusion Processing of Carbohydrate Sources on Weanling Pig Performance 1,2 Introduction", Journal Animal Science, vol. 76 1 January 1998 (1998-01-01), pages 3034-3042, XP055408626, Retrieved from the Internet: URL:https://www.animalsciencepublications.org/publications/jas/pdfs/76/12/3034?search-result=1 [retrieved on 2017-09-21] * the whole document * -----	1-7	
A	CN 103 005 160 A (UNIV ZHEJIANG) 3 April 2013 (2013-04-03) * the whole document * -----	1-7	

TECHNICAL FIELDS SEARCHED (IPC)

The present search report has been drawn up for all claims

Place of search	Date of completion of the search	Examiner
The Hague	25 September 2017	Heirbaut, Marc

4

CATEGORY OF CITED DOCUMENTS

X : particularly relevant if taken alone
Y : particularly relevant if combined with another document of the same category
A : technological background
O : non-written disclosure
P : intermediate document

T : theory or principle underlying the invention
E : earlier patent document, but published on, or after the filing date
D : document cited in the application
L : document cited for other reasons
& : member of the same patent family, corresponding document

EPO FORM 1503 03.82 (P04C01)

page 2 of 2

ANNEX TO THE EUROPEAN SEARCH REPORT ON EUROPEAN PATENT APPLICATION NO. EP 17 00 0914

This annex lists the patent family members relating to the patent documents cited in the above-mentioned European search report. The members are as contained in the European Patent Office EDP file on
The European Patent Office is in no way liable for these particulars which are merely given for the purpose of information.

25-09-2017

Patent document cited in search report	Publication date	Patent family member(s)	Publication date
CN 103005160 A	03-04-2013	NONE	

EPO FORM P0459

For more details about this annex : see Official Journal of the European Patent Office, No. 12/82
