# Supplementary material for: Soft-Pellet Feed Strategy for Broilers: Effects on Growth Performance and Liver–Intestinal Health
Source: Animals (Basel). 2026 Jul 26;16(15):2308. doi: 10.3390/ani16152308 (PMC13463713; doi:10.3390/ani16152308)
Supplement: Supplementary file 1 [file animals-16-02308-s001.zip › Supplementary Table S1.pdf]

Supplementary Table S1. Effect of feed types on α-diversity in cecum microbiota of broilers.

**Items**	**CON**	**TG1**	**TG2**	**TG3**	**SEM**	***P* value**
Stage 1 (1-21 d)						
Ace	294.93	285.82	282.90		7.15	0.789
Chao1	302.12	289.60	289.89		7.09	0.731
Shannon	3.79	3.64	3.63		0.72	0.638
Simpson	0.06	0.07	0.07		0.01	0.878
